# Polyelectrolyte Copolymer Nanoreactors: From Colloidal Assembly to Photoredox Activity in Water

**DOI:** 10.1021/acsami.6c00473

**Published:** 2026-03-16

**Authors:** Afshin Nabiyan, Mitra Esfandiari, Sergio Kogikoski, Ilko Bald, Evgenii Titov, Michael U. Kumke, Nora Kulak, Helmut Schlaad

**Affiliations:** Institute of Chemistry, 26583 University of Potsdam, Karl-Liebknecht-Str. 24-25, Potsdam 14476, Germany

**Keywords:** copolymers, polyelectrolyte, self-assembly, photoredox, water, nanoreactors

## Abstract

Performing organic photoredox reactions in water remains challenging because most catalysts cannot simultaneously solubilize substrates, control nanoscale organization, and maintain activity under aqueous conditions. We report a photoredox-active polyelectrolyte based on a polydehydroalanine (PDha) backbone covalently functionalized with polypyridyl complexes to address some of these limitations. The copolymer undergoes substrate-triggered self-assembly in water, forming photocatalytically active spherical colloidal nanostructures (∼30 nm), as confirmed by dynamic light scattering (DLS) and transmission electron microscopy (TEM). The assemblies efficiently catalyze the hydroxylation of arylboronic acids, a representative water-insoluble photoredox transformation. Mechanistic studies using UV-visible spectroscopy, Raman spectroscopy, time-resolved emission spectroscopy, electrochemistry, and density functional theory (DFT) indicate that dual hydrogen bonding between PDha carboxylates and arylboronic acids governs both self-assembly and catalytic performance. The nanostructures retain high activity over multiple cycles. These findings establish adaptive polymer self-assembly as a general strategy for creating enzyme-like, water-compatible photoredox systems and provide a platform for transferring organic photoredox chemistry into aqueous media.

## Graphical abstract

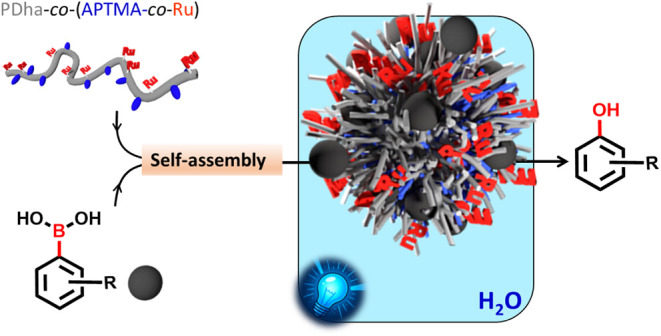

## Introduction

Supramolecular strategies have recently driven significant advances in photoredox catalysis.^1–3^ These approaches have opened new opportunities for conducting organic transformations in aqueous media.^2,4,5^ Nevertheless, water-based photoredox catalysis remains underdeveloped relative to nonaqueous systems due to the challenge of designing photocatalysts that remain stable and efficient in complex aqueous environments.^6–9^ Consequently, reactions conducted directly in pure water are still relatively rare and often difficult to achieve.^9^

Key challenges in aqueous photoredox catalysis include the poor solubility of reactants, catalysts, and photocatalysts, limited scalability, and instability of reactive intermediates. Additional complications arise from limited light penetration, catalyst degradation under prolonged irradiation, side reactions with water, and inefficient substrate–catalyst–light interactions.^2,4,8,10–14^ To address these problems, various water-soluble dye sensitizers and metal complexes, such as iridium and ruthenium complexes,^15^ Eosin Y,^16,17^ Rose Bengal,^18^ and methylene blue^17^ have been explored. Nevertheless, even with water-soluble photoredox catalysts in hand, solubility limitations persist for many catalytically relevant species, including substrates and electron mediators. Structural modifications that improve the solubility of photoredox catalysts often perturb excited-state lifetimes or shift redox potentials, ultimately reducing catalytic efficiency. Achieving a balance between water solubility and optimal photophysical performance, therefore, remains a major limitation, confining many efficient photocatalysts to organic solvents.^19,20^ Improving aqueous compatibility typically requires complex ligand modifications or multistep synthetic routes, which are demanding and not broadly applicable.^2,11,15,19,21^ Recently, supramolecular photoredox chemistry, drawing inspiration from natural photosynthetic systems, has emerged as a promising strategy to overcome these limitations.^1,2,10,22^ By combining covalent and noncovalent design principles, such systems can enhance solubility in water, and even improve catalytic activity, and expand the reaction scope.^1,2,10^

Among the various supramolecular strategies, polymer-based scaffolds provide distinct advantages for constructing stable and well-defined catalytic microenvironments.^10,23–27^ Unlike low molecular weight amphiphiles, i.e., surfactants, which are sometimes employed in photoredox reactions but lack robust catalytic environments,^2,7,13,27,28^ polymeric assemblies form persistent nanoscale compartments with high structural diversity. Indeed, surfactants can serve as simple model systems to probe photoredox reactivity, but they often exhibit high critical micelle concentrations and limited stability under reaction conditions, varying concentrations, or during purification.^13,25,29,30^ In contrast, polymeric assemblies benefit from strong interblock interactions and chain entanglement, as well as compositional and architectural tunability, enabling high guest-loading capacity, improved thermal stability, and complex designs.^23,31–33^ Consequently, polymer-based scaffolds constitute versatile and reliable platforms for catalysis, and could thus offer more precise control over substrate–catalyst interactions in self-assembled systems.^23,34–36^

By combining polymer chemistry with supramolecular design, a wide range of catalytic environments can be accessed, including micelles, vesicles, hydrogels, dendrimers, interpolyelectrolyte complexes, colloidal nanoparticles, polymer-grafted inorganic nanoparticles, and single-chain polymer nanoparticles (SCNPs).^10,31,37–40^ Each class exhibits distinct advantages and limitations. Micellar assemblies, for example, provide well-defined hydrophobic cores that efficiently solubilize and concentrate hydrophobic substrates and catalysts.^23,24,31,41–43^ Hydrogels, formed from cross-linked polymer networks, offer robust three-dimensional scaffolds that enable controlled substrate diffusion and facilitate catalyst recovery.^44^ Dendrimers, with their highly branched architectures, allow multivalent interactions and tunable microenvironments for catalysis.^45^ Colloidal nanoparticles and polymer-grafted inorganic nanoparticles combine high surface areas with excellent colloidal stability.^16,34,42^ SCNPs provides precise control over polymer composition.^10,26^ Despite these advances, significant challenges remain that limit the broader applicability of SCNP systems.^40^

Photoredox-active units can be incorporated into polymeric frameworks either physically, through encapsulation within amphiphilic domains such as micelles or polyelectrolyte complexes, or covalently via postpolymerization conjugation or copolymerization with functional monomers, as demonstrated in SCNPs and colloidal nanoparticles.^34,40,41,46^ Covalent attachment, typically to the polymer backbone, provides greater structural stability and minimizes catalyst leaching, even under dilute conditions. In both approaches, the conformational flexibility and spatial organization of the polymer scaffold create well-defined microenvironments that enhance catalytic performance.^14,22,47^ Most polymeric and amphiphilic scaffolds, once functionalized with catalytic segments, primarily act as passive supports. They stabilize photoredox or catalytic components through encapsulation, create hydrophobic pockets, prevent aggregation, and provide microenvironments but rarely interact directly with substrates or participate actively in the catalytic cycle. Truly dynamic polymers capable of substrate sensing or adaptive modulation of conformation or electronic properties during catalysis remain rare.^1,2,48^ Consequently, these materials often function as inert hosts rather than smart catalytic platforms, leaving much of the self-assembly and functional-group engineering potential of polymers underexploited in aqueous photoredox systems.^10,22,25^ In this context, recent advances with SCNPs and stimuli-responsive (co)­polymers demonstrate progress toward more interactive polymer-based catalysis and greater scaffold engagement.^26,49^ However, their practical utility remains limited. For instance, catalysis in SCNPs often relies on nonspecific polarity matching rather than molecular recognition, reducing selectivity^10,26,40^ Random intramolecular cross-linking and the absence of sequence control complicate predictable active-site organization, and the compact SCNP topology can restrict substrate diffusion. Their performance frequently requires ultradilute conditions, limiting scalability and reproducibility, while incomplete understanding of folding and morphology hinders the formation of well-defined catalytic sites.^10,26,40^

Among various polymeric systems, polyelectrolytes have been explored as particularly attractive scaffolds for constructing photoredox supramolecular architectures.^34,35,44^ Building on these principles, Schacher et al.^16,34^ developed a polydehydroalanine (PDha)-based copolymer capable of forming photoredox assemblies. Polyelectrolytes, charged macromolecules bearing cationic, anionic, or zwitterionic groups along their backbone or side chains, have been widely used to support photoredox transformations.^50,51^ PDha, introduced as a polyampholyte, facilitates hydrogen production by incorporating hydrophobic chromophores such as perylene monoimide and boron-dipyrromethene (BODIPY).^34,52^ The ampholytic nature of the PDha backbone enables strong interactions with a broad range of species, including dyes,^34,52^ nanoparticles,^16^ and carbon nanotubes.^53^ While these polyelectrolyte-based and other polymeric assemblies demonstrate the potential of polymer scaffolds, controlling catalyst organization and sustaining efficient photoredox cycles in water remain major challenges. Addressing these limitations calls for adaptive, modular polymer platforms that provide stable microenvironments and can actively modulate catalysis, enhancing rates, selectivity, and substrate accessibility while managing phase behavior, light penetration, and transient excited states.^2,4,9–11,51^

Here, we introduce a smart, adaptive PDha-based copolymer covalently functionalized with Ru­(II) polypyridyl complexes that undergoes reactant-induced self-assembly into colloidal nanostructures. Dynamic light scattering (DLS) and transmission electron microscopy (TEM) confirm the formation of spherical nano-objects with diameters of approximately 30 nm. Using visible-light-driven hydroxylation of arylboronic acids as a benchmark, these nanostructures demonstrate high catalytic efficiency, robustness, and recyclability. Raman spectroscopy, time-resolved emission spectroscopy, and density functional theory (DFT) explain the formation of these colloidal assemblies, revealing that dual hydrogen-bonding interactions between the polymer and arylboronic acid substrates are the major driving force. This work illustrates that substrate-responsive polymer assembly provides a versatile strategy for designing water-compatible photoredox catalysts with a tunable structure, adaptive behavior, and the potential to overcome challenges in controlling catalyst organization and activity in aqueous media.

## Experimental Section

### Chemicals and Materials

1a,9b-Dihydrooxireno­[2,3-*f*]­[1,10]­phenanthroline (DHPH, ≥98%), (*p*-methoxyphenyl)­boronic acid (≥95.0%), *p*-nitrophenylboronic acid (≥95.0%), 4-formylbenzeneboronic acid (≥95.0%), 4-boronobenzoic acid (≥98.0%), *p*-fluorophenylboronic acid (≥95%), 4-ethylbenzeneboronic acid (≥98.0%), 2,6-dimethylbenzeneboronic acid (≥95%), methanesulfonyl chloride (≥99.7%), triethanolamine (≥98%), (3-acryl amidopropyl)­trimethylammonium chloride solution (APTMA), 75 wt % in H_2_O), *cis*-bis­(2,2′-bipyridine)­dichlororuthenium­(II) (≥97%), and 2,3,4,6,7,8,9,10-octahydropyrimidol­[1,2-*a*]­azepine (DBU) (≥98%) were purchased from Sigma-Aldrich (USA). Trifluoroacetic acid (TFA) (≥99.9%) and NaOH (0.1 N) were purchased from Roth (Karlsruhe, Germany), triethylamine (≥99.0%) was purchased from CHEMSOLUTE (Renningen, Germany), and *N*-(*tert*-butoxycarbonyl)-l-serine methyl ester (98%) was purchased from Carbolution Chemicals (St. Ingbert, Germany). Organic solvents (Schwerte, Germany), 5-formyl-2-methoxybenzeneboronic acid (≥97%), 2-fluoro-4-methoxybenzeneboronic acid (≥97%), and 4-(hydroxymethyl)­benzeneboronic acid (≥97%) were purchased from TCI chemicals (Tokyo, Japan). HCl (37% solution) was purchased from Fischer Scientific (Hampton, USA). *N,N*-Diisopropylethylamine (*i*Pr_2_NEt) (≥95%) was purchased from Fluka Analytical.

Detailed descriptions of polymer synthesis, and characterization and photocatalysis reactions are provided in the Supporting Information.

## Analytical Instrumentation

### Nuclear Magnetic Resonance (NMR) Spectroscopy

^1^H and ^13^C NMR spectra were recorded on Bruker 300 and 400 MHz spectrometers at a temperature of 25 °C. The solvents used for these spectra included MeOD (methanol-d_4_), CDCl_3_ (chloroform-d), DMSO-*d*_6_ (dimethyl sulfoxide-d_6_), and D_2_O/NaOD (deuterated water with sodium deuteroxide). The spectra were referenced by using the residual signal of the respective deuterated solvent.

### Size Exclusion Chromatography (SEC)

SEC measurements were conducted using specialized instruments suited for different solvent systems. In THF, an Agilent 1260 Infinity System was employed, featuring a 1260 IsoPump (G1310B), a 1260 ALS autosampler (G1310B), and three consecutive PSS SDV columns (5 μm, 8 × 300 mm). The system operated at a flow rate of 1 mL min^–1^ with columns heated to 30 °C. Detection was achieved using an Agilent 1260 DAD VL (GG1329B) for UV–vis analysis and a 1260 RID (G1315D) refractive index detector. For measurements in aqueous conditions, a Jasco system from Groß-Umstadt, Germany, was utilized, featuring a PU7980 pump and an RI72031 Plus refractive index detector. The solvent consisted of water with 0.3% trifluoroacetic acid (TFA) and 0.1 M NaCl, flowing at 1 mL min^–1^ through a PSS SUPREMA 30 Å column maintained at 30 °C.

### Dynamic Light Scattering (DLS)

DLS experiments were performed using a Zetasizer Ultra instrument from Malvern Panalytical Ltd. (Malvern, U.K.) at a temperature of 25 °C. The measurements were conducted in disposable polystyrene cuvettes, and each sample was equilibrated for 10 min before measurement to ensure temperature stability.

### UV–Vis Spectroscopy (UV/Vis)

UV–vis measurements were performed by using an Agilent Cary 60 spectrometer to determine the absorbance characteristics of the samples. The samples were placed in a Hellma quartz glass cuvette with a path length of 10 mm, ensuring minimal interference from the container. Measurements were conducted at room temperature, and the absorbance spectra were recorded over a wavelength range of 200 to 800 nm with a resolution interval of 5 nm.

### Thermogravimetric Analysis (TGA)

Thermogravimetric analysis (TGA) was conducted by using a PerkinElmer TGA800 device to assess the thermal stability and composition of the samples. The analysis was carried out under a continuous airflow to ensure an oxidative environment, which helps to complete decomposition of the sample. The samples were heated from 30 to 850 °C at a controlled heating rate of 10 K min^–1^.

### Transmission Electron Microscopy (TEM)

Samples were examined by using a JEOL JEM-1011 transmission electron microscope (JEOL, Akishima, Tokyo, Japan) equipped with an Olympus MegaView G2 camera and operated at an accelerating voltage of 80 kV. Imaging was performed on copper grids coated with a 1 nm carbon layer on top of a 10 nm Formvar film (EFCF400-Cu-50, Science Services GmbH, Munich, Germany). To improve sample adhesion and remove organic residues, grids were plasma-cleaned for 15 s using a Diener Electronic Zepto plasma cleaner prior to sample application. Subsequently, 5 μL of colloidal nanoaggregate solution was applied to the grids and allowed to incubate for 2–3 min before analysis.

### Gas Chromatography–Mass Spectrometry (GC-MS)

GC-MS measurements were conducted with an HP 6890 Series Gas Chromatograph (Hewlett-Packard) connected to an Agilent Technologies 5973 Network Mass Selective Detector (MSD). Samples were first separated by the GC system and then analyzed by electron ionization (EI) and time-of-flight (TOF) mass spectrometry. Both low- and high-resolution mass spectra were recorded under the EI/TOF conditions. Data were acquired and processed using the GCMS5973 Data Analysis software, with additional support from Enhance Data Analysis tools for interpretation.

### Electrochemical Measurements

Cyclic voltammetry (CV) measurements were carried out using a μStat 400 Bipotentiostat/Galvanostat (Metrohm DropSens), with data acquisition and analysis managed through DropView 8400 software. Experiments were performed in an aqueous medium containing 100 mM KCl as the supporting electrolyte. The electrochemical setup utilized a screen-printed carbon electrode (SPCE), Metrohm DropSens 11L, featuring a carbon working electrode, a carbon counter electrode, and an integrated Ag/AgCl reference electrode on a ceramic base. Cyclic voltammograms were collected within a potential window from −0.6 V to +1.2 V versus Ag/AgCl, using scan rates between 25 mV·s^–1^ and 500 mV·s^–1^. Each measurement commenced at +0.1 V, with the initial scan proceeding in the anodic (positive) direction. Throughout all experiments, the analyte concentration was kept constant at 1.5 mM.

### Raman Spectroscopy

Raman measurements were carried out using a LabRAM HR-Evolution microscope (Horiba Jobin-Yvon, France) equipped with a 785 nm laser (∼7 mW cm^–2^ at the focal plane). The laser was focused onto the sample using a 100× objective (N.A. 0.90, Olympus). Each spectrum was recorded following approximately 1 h of continuous irradiation.

### Time-Resolved Emission Spectroscopy

A laser system consisting of a pulsed Nd:YAG laser (Spectra-Physics, Quanta-Ray LAB 170, USA) with a repetition rate of 20 Hz and an optical parametric oscillator (OPO, GWU Lasertechnik, Germany) was used. With an iCCD camera (Andor Technology, DH720–18H-13, United Kingdom) combined with a spectrograph (Shamrock 303i with 300 grooves/500 nm blaze grating, Ireland), the luminescence emission was detected. The time-resolved Ru- luminescence (excitation wavelength λ_ex_ = 450 nm) was measured by using the boxcar technique. After a 75 ns initial delay time, 100 emission spectra (50 accumulations each) with a constant time step of Δt = 50 ns were measured to record a full luminescence decay kinetics. A gate width of 4 μs was applied. The luminescence was detected in the spectral range of 575 nm < λ_em_ < 710 nm.

### Quantum Chemical Calculations

Details can be found in the Supporting Information.

## Results and Discussion

### Synthesis and Characterization of PDha Copolymer

A water-soluble PDha copolymer carrying covalently attached Ru­(II)-pyridyl complexes was constructed using a modular stepwise grafting strategy (Figure 1A), adapted from previous work.^34,35,54^ In the first step, nitroxide-mediated radical polymerization (NMP) of methyl 2-((*tert*-butoxycarbonyl)­amino)­acrylate (M*t*BAMA) afforded PM*t*BAMA with an apparent number-average molecular mass (*M*_n_) of 70.0 kg mol^–1^ and a dispersity (*Đ*) of 1.5, as determined by size exclusion chromatography (SEC, eluent: THF) with PMMA calibration. Subsequent deprotection with trifluoroacetic acid (TFA) exposed primary amine groups along the PDha chain, with an average of 180 repeating units (based on monomer conversion), thereby providing reactive sites for postpolymerization modification (Figure 1A). In this way, the type, efficiency, and degree of grafting onto the amino groups of the PDha backbone can be precisely controlled by adjusting the reaction time and reaction conditions, as reported previously.^54^

**Figure 1:**
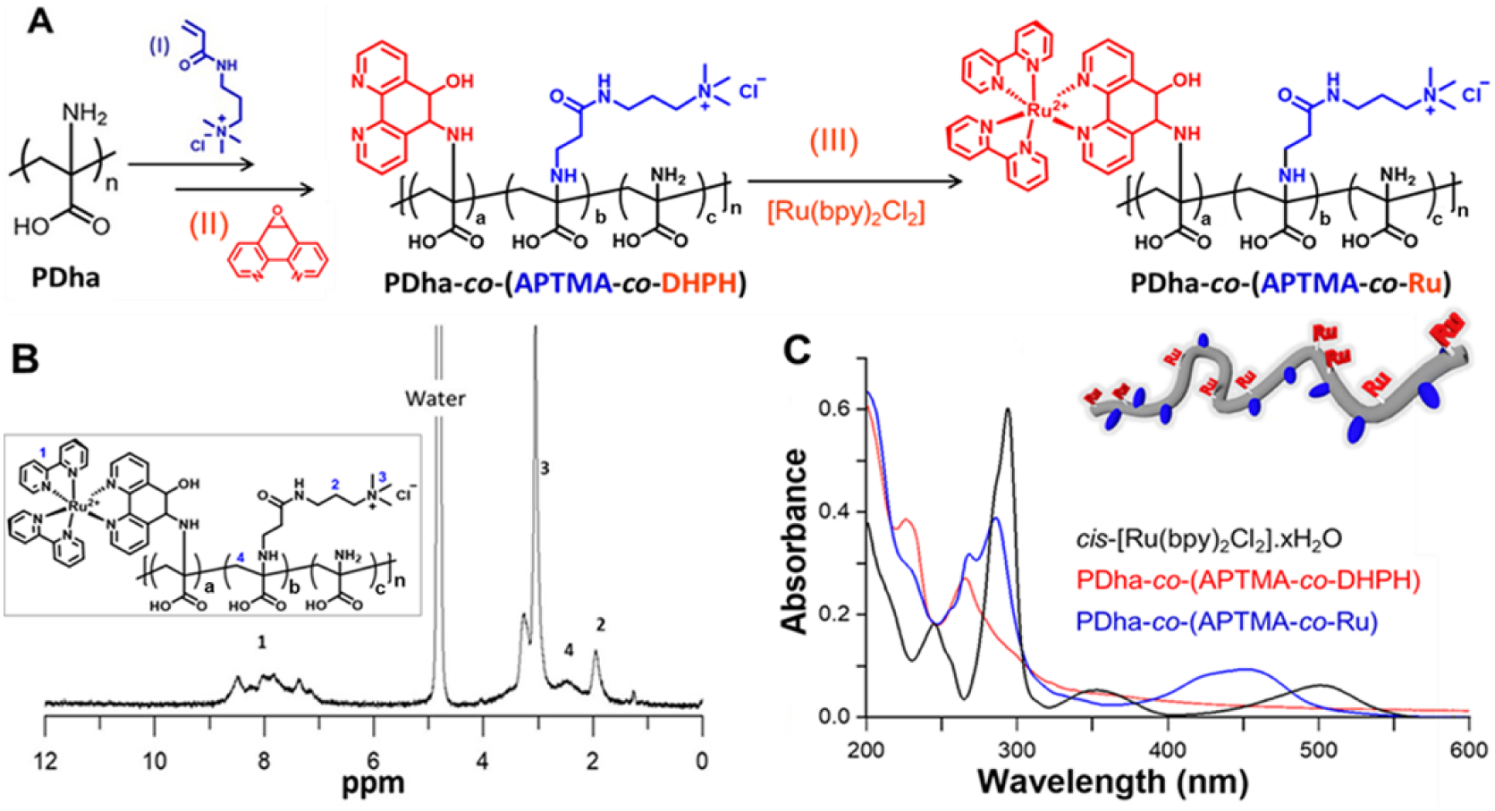
(A) Schematic depiction of the synthetic route toward ruthenium-containing PDha-based copolymer (a = 0.1, b = 0.4, and c = 0.5). (B) ^1^H NMR spectrum of PDha-*co*-(APTMA-*co*-Ru) in D_2_O/NaOD. (C) UV–vis absorption spectra of PDha-*co*-(APTMA-*co*-DHPH) (red, 0.005 mg/mL) and different Ru complexes: PDha-*co*-(APTMA-*co*-Ru) (blue, 0.005 mg/mL) in water and *cis*-bis­(2,2′-bipyridine)­dichlororuthenium­(II) [Ru­(bpy)_2_Cl_2_] in acetonitrile (black, 0.005 mg/mL).

In the next step, (3-acrylamidopropyl)­trimethylammonium chloride (APTMA) side chains were grafted onto PDha via aza-Michael addition to form PDha-*co*-APTMA (Figure 1A, step I). An estimated grafting density of approximately 40% was obtained from^1^H NMR spectroscopy by integrating the APTMA-derived proton signals and comparing them to the PDha backbone protons (Figure S2).^54^ Afterward, the (water-soluble) PDha-*co*-APTMA was further functionalized with 1a,9b-dihydrooxireno­[2,3-*f*]­[1,10]­phenanthroline (DHPH) via epoxide ring opening to introduce Ru­(II) coordination sites (Figure 1A, step II), as evidenced by the appearance of aromatic signals in the ^1^H NMR spectrum (Figure S4). Subsequent complexation with a Ru­(II)-pyridyl precursor in EtOH/H_2_O afforded the final conjugate, PDha-*co*-(APTMA-*co*-Ru) (Figure 1A, step III). An estimated Ru complex incorporation of ∼10% was obtained by ^1^H NMR spectroscopy, based on comparing the integrals of the newly appearing Ru–pyridyl aromatic resonances with the PDha backbone protons (Figures 1B and S5).^54^

To further probe the coordination environment and photophysical properties of the Ru-functionalized polymer, a combination of analytical and computational techniques was employed, including density functional theory (DFT) calculations, thermogravimetric analysis (TGA), and UV–vis absorption spectroscopy. TGA revealed a distinct decomposition profile for PDha-*co*-(APTMA-*co*-Ru), with two major weight-loss events near 250 °C and 650 °C, and a notably high residual mass (∼50 wt %), likely corresponding to stable Ru-containing residues such as ruthenium oxides (Figure S6).

UV–vis spectroscopy was then used to probe the optical properties of the Ru complexes before and after conjugation to the polymer backbone (Figures 1C and S7). Generally, the parent complex [Ru­(bpy)_2_Cl_2_] exhibits several characteristic absorption bands: metal-to-ligand charge transfer (MLCT) transitions centered at 502 and 360 nm, and a ligand-centered transition at 295 nm. The polymer precursor PDha-*co*-(APTMA-*co*-DHPH) exhibited a strong absorption at around 267 nm, attributable to the phenanthroline moieties, with no features in the visible region. Upon Ru coordination, a pronounced hypsochromic MLCT characteristic peak appeared in the spectrum of PDha-*co*-(APTMA-*co*-Ru) at ∼451 nm, reflecting an altered electronic environment around the Ru center compared to [Ru­(bpy)_2_Cl_2_]. Furthermore, the absorption feature at λ = 352 nm, present in the free [Ru­(bpy)_2_Cl_2_] complex, disappeared in the polymer-conjugated form, indicating significant perturbation of ligand-centered transitions. Additional absorptions corresponding to bipyridine and phenanthroline units were retained in the PDha-*co*-(APTMA-*co*-Ru) spectrum, confirming the successful incorporation of the Ru­(II) chromophores. To rationalize the observed spectroscopic changes, we performed time-dependent DFT (TD-DFT) calculations (at the B3LYP+D3­(BJ)/def2-SVP/CPCM level as implemented in Orca 6)^55^ using representative polymer fragments as models. The calculated spectra closely matched the experimental observations (Figure S7). Charge density difference (CDD) plots confirmed that the major transitions in the visible region are MLCT in nature (Table S2). Notably, in [Ru­(bpy)_2_Cl_2_], the chloride ligands contributed to the hole density, supporting the spectral differences observed upon polymer conjugation.

### Solution and Self-Assembly Behavior of Copolymers

Both PDha-*co*-APTMA and PDha-*co*-(APTMA-*co*-Ru) gave clear aqueous solutions, with the latter exhibiting a deep orange color characteristic of Ru­(II) complexes (Figure 2A). Dynamic light scattering (DLS) analysis showed that PDha-*co*-APTMA was molecularly dissolved as unimers in aqueous solution, and Ru incorporation led to a modest increase in the hydrodynamic diameter (4–8 nm, Figure S8). To investigate substrate-induced changes in copolymer organization and establish a model system for reaction-condition optimization, 4-methoxyphenylboronic acid (4-MPBA), a simple water-insoluble hydrophobic arylboronic acid, was chosen as the initial reactant. Upon addition to PDha-*co*-(APTMA-*co*-Ru), 4-MPBA triggered polymer self-assembly and the formation of colloidal nanoaggregates, likely driven by strong noncovalent interactions between the polymer and substrate (Scheme S1). Its clean reaction profile and ease of product analysis made 4-MPBA a convenient model substrate. This behavior motivated a detailed investigation of the aggregation mechanism using visual inspection, DLS, transmission electron microscopy (TEM), Raman spectroscopy, and ground-state DFT calculations.

**Figure 2:**
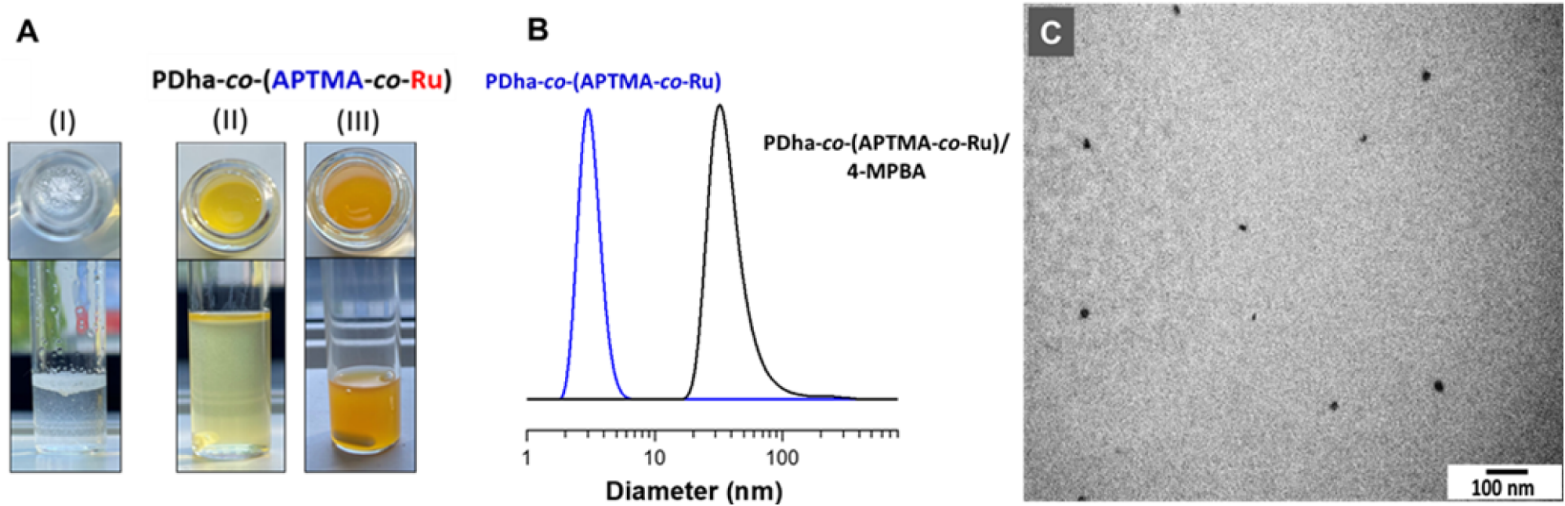
(A) Optical photographs of 4-MPBA in water (I, left) and PDha-*co*-(APTMA-*co*-Ru)/4-MPBA dispersions at 0.1 mg/mL (II, middle) and 1 mg/mL (III, right). (B) DLS size distribution by intensity for the PDha-*co*-(APTMA-*co*-Ru) copolymer and PDha-*co*-(APTMA-*co*-Ru)/4-MPBA mixtures at concentrations of 0.1 mg/mL (PDha-*co*-(APTMA-*co*-Ru)) and 0.01 mg/mL (4-MPBA). (C) TEM images of PDha-*co*-(APTMA-*co*-Ru)/4-MPBA (4-MPBA with a concentration of 0.2 mg/mL and PDha-*co*-(APTMA-*co*-Ru) with a concentration of 1 mg/mL).

Our initial visual observations revealed that 4-MPBA and most of the arylboronic acid substrates are not well soluble in water and undergo solid–liquid phase separation, resulting in the formation of a macroscopic white precipitate (Figure 2A (I)). In sharp contrast, when combined with PDha-*co*-(APTMA-*co*-Ru), a marked change in behavior was observed: the mixture formed a light-orange homogeneous dispersion, which, even at low polymer concentrations, appeared as a uniform yellow dispersion (Figure 2A (II and III)). The obtained dispersions were subjected to DLS analysis, which revealed a substantial increase in hydrodynamic diameter from ∼4–8 nm to ∼30 nm upon the addition of 4-MPBA, confirming the formation of aggregates (Figures 2B and S8). TEM analysis of drop-cast samples (PDha-*co*-(APTMA-*co*-Ru)/4-MPBA = 1:0.2, wt/wt) showed collapsed spherical aggregates with diameters of 16–35 nm (Figures 2C and S9).

The remarkable solubilization accompanied by nanoaggregate formation provides compelling evidence of well-defined intermolecular interactions between the hydrophilic polymer scaffold and the poorly water-soluble aromatic substrate. Therefore, ground-state DFT calculations (B3LYP+D3­(BJ)/def2-SVP/CPCM­(water)) were used to reveal hydrogen bonding interactions between the boronic acid group of 4-MPBA and the carboxylic acid moieties of the PDha backbone. This interaction forms a robust, energetically favorable binding motif that persists in both protonated and deprotonated states (Tables S3–S4, Figure 4A). This behavior is consistent with previous reports of PDha copolymers interacting with diverse hydrophobic systems.^34,53^ Interestingly, such stabilization occurs independently of the Ru and APTMA units (Table S5), suggesting that the PDha chains alone are sufficient to mediate substrate association. Experimentally, this interaction manifests in the formation of colloidally stable nanoaggregates, supposedly driven by a synergy of hydrogen bonding, electrostatic complementarity, and π–π stacking between aryl groups and the Ru complex. These nanostructures, visually evident through increased turbidity and dispersion behavior (Figure 2A and B), likely function as compartmentalized reaction domains, concentrating both catalyst and reactants in water and facilitating an effective photocatalytic process.

### Photocatalytic Conversion of Arylboronic Acids to Phenols

Phenols are important compounds with broad applications in chemical and pharmaceutical industries due to their diverse biological activities, including antioxidant and anticancer effects. Leveraging the stability of boronic acid derivatives, photoredox catalysis enables an efficient and selective hydroxylation of arylboronic acids to phenols under mild visible-light conditions.^56–59^ To evaluate the photocatalytic activity of PDha-*co*-(APTMA-*co*-Ru), we selected this visible-light-driven hydroxylation of arylboronic acids to phenols, a benchmark reaction first reported by Xiao’s group (Figure 3A, Table 1).^60^ Light irradiation of a mixture containing only PDha-*co*-(APTMA-*co*-Ru) and 4-MPBA in water resulted in no detectable conversion (Table 1, entry 1). However, upon the addition of a base to the same mixture (Table 1, entries 2–4), the formation of 4-methoxyphenol was observed. Among the tested bases, *N*,*N*-diisopropylethylamine (*i*Pr_2_NEt, 0.05 M) led to the highest conversion, exceeding 95% (Table 1, entry 4). Notably, conversion was determined by ^1^H NMR spectroscopy, considering the signals of hexamethyldisiloxane (HDMSO, internal standard) and the methoxy resonances of 4-MPBA (δ 3.80 ppm, CD_3_OD) and the 4-methoxyphenol product (δ 3.73 ppm, CD_3_OD) (Figure 3A).

**Table 1:**
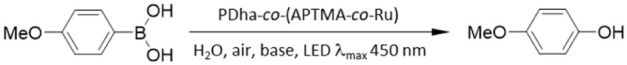
Screening and Control Experiments of Hydroxylation of 4-MPBA to 4-Methoxyphenol by PDha-*co*-(APTMA-*co*-Ru)^a^

Entry	Catalyst (a)	Base (0.05 mM)	Con. (%) ^(d)^
1	PDha-*co*-(APTMA-*co*-Ru)	-	<0.5
2	PDha-*co*-(APTMA-*co*-Ru)	Et_3_N	∼60
3	PDha-*co*-(APTMA-*co*-Ru)	NaOH	∼90
4	PDha-*co*-(APTMA-*co*-Ru)	*i*Pr_2_NEt	>95
5	PDha-*co*-(APTMA-*co*-Ru)^b^	*i*Pr_2_NEt	0
6	PDha-*co*-APTMA^c^	*i*Pr_2_NEt	0
7	PDha-*co*-(APTMA-*co*-DHPH)^c^	*i*Pr_2_NEt	0

a PDha-*co*-(APTMA-*co*-Ru) was used as the polymeric photocatalyst under air in a 2 mL reaction mixture. The catalyst concentration was set to 0.5 mg of polymer per mL, and 4-MPBA was introduced as the substrate at 0.01 mM. The reaction mixture was irradiated with 450 nm LED light (λ_max_) for 24 h. Conversion was quantified directly from the crude mixture by ^1^H NMR spectroscopy. Hexamethyldisiloxane (HMDSO) served as an internal standard, and the HMDSO signal was compared to the −OMe resonance of 4-MPBA (0.03 mM) for quantification.

b The reaction was also performed under dark conditions.

c Control experiments were carried out using PDha-*co*-APTMA or PDha-*co*-(APTMA-*co*-DHPH) with a total concentration of 0.5 mg/mL, and 4-MPBA was introduced as the substrate at 0.01 mM.

**Figure 3:**
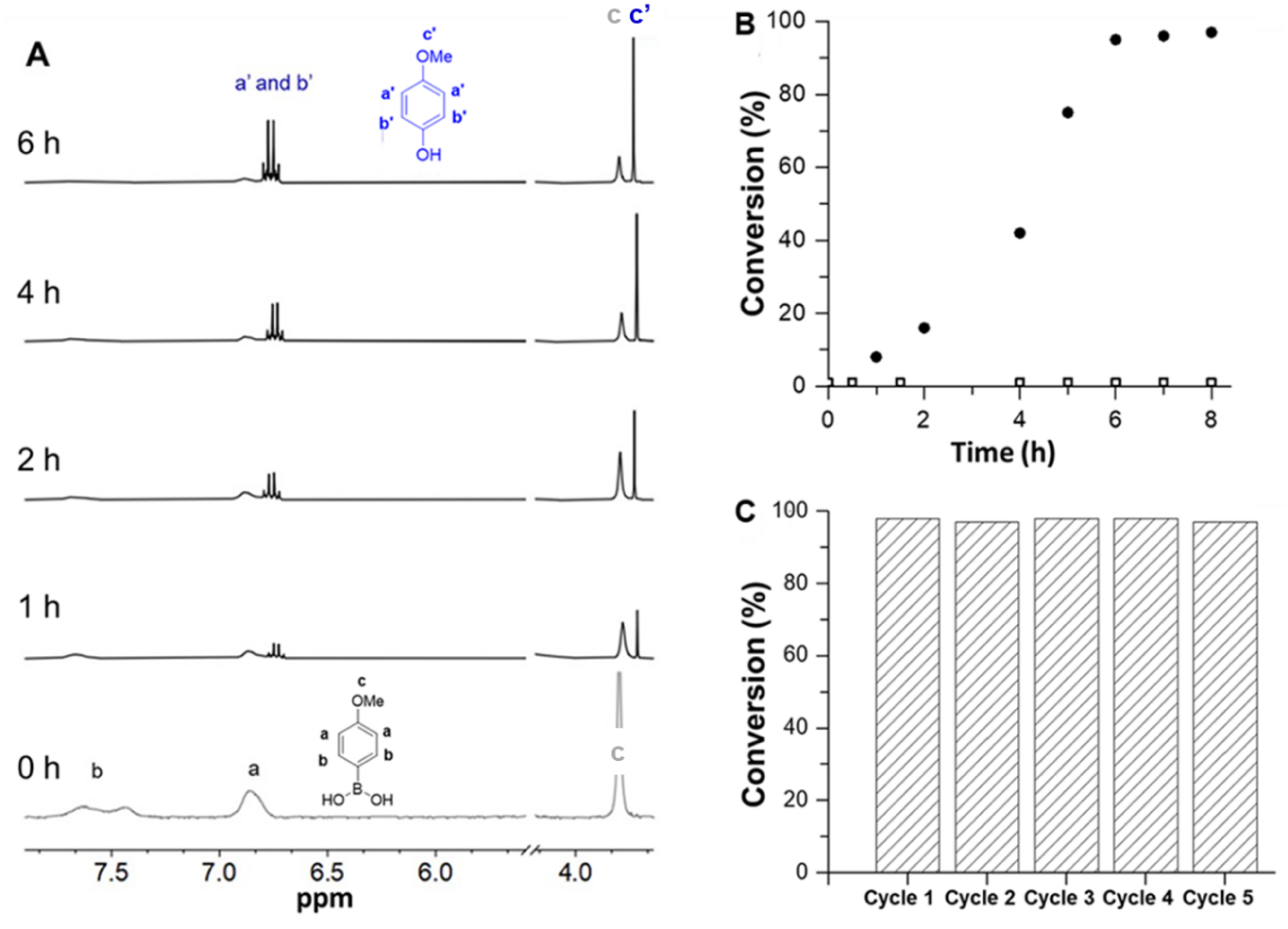
(A) Representative ^1^H NMR (400 MHz) spectra of the conversion of 4-MPBA to the corresponding phenol product at different time intervals (under conditions corresponding to Table 1, entry 4). (B) Conversion vs time plot of 4-MPBA corresponding to Table 1, entry 4: (• under irradiation, λ = 450 nm, and entry 5 □ dark). (C) Recyclability of photocatalyst PDha-*co*-(APTMA-*co*-Ru) under conditions corresponding to Table 1, entry 4.

To understand the contribution of each component, control experiments were performed, which showed that the catalyst (i.e., PDha-*co*-(APTMA-*co*-Ru)), base, and light are all necessary for reactivity. Omission of any single element resulted in complete suppression of product formation (Table 1, entries 1 and 5–7, and Figure 3B). After optimizing the reaction parameters, we observed the conversion of 4-MPBA to 4-methoxyphenol over time (Figure 3B). Our results show that the conversion reaches its maximum after nearly 7 h (Figure 3B).

The reusability of PDha-*co*-(APTMA-*co*-Ru) was also evaluated by repeated cycles of oxidative hydroxylation of 4-MPBA (Figure 3C). After 7 h of irradiation, the photocatalyst was recovered by ethyl acetate extraction, and the residual ethyl acetate solvent was removed under vacuum before reuse. The catalyst maintained high activity over five consecutive cycles, consistently yielding phenol in >95%. Structural integrity was monitored by in situ ^1^H NMR spectroscopy before and after cycling (Figure S10) directly from the reaction mixture. However, new signals emerged in the PDha backbone and aromatic regions after several hours of running the reaction, likely due to the formation of side products and perhaps a minor Ru ligand detachment. Despite these changes, the consistently high yields underscore the catalyst’s chemical and photochemical stability.

### Exploring the Scope of Hydroxylation by PDha-*co*-(APTMA-*co*-Ru)

With the optimized conditions in hand (see Table 1), we evaluated the generality of PDha-*co*-(APTMA-*co*-Ru) as a photocatalyst for the oxidative hydroxylation of a broad range of arylboronic acids (Table 2, entries 1–9). The transformation proceeded efficiently across diverse substrates, affording phenols in good to excellent yields. Electron-donating groups, such as methyl and propyl (entries 1 and 2), furnished phenol products in >90% conversion. Notably, sensitive functional groups, including aldehyde and hydroxyl, were well tolerated, delivering phenols in >90% (entries 3 and 4).

**Table 2:**
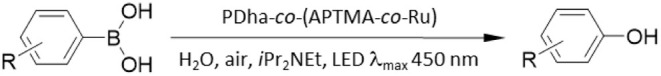

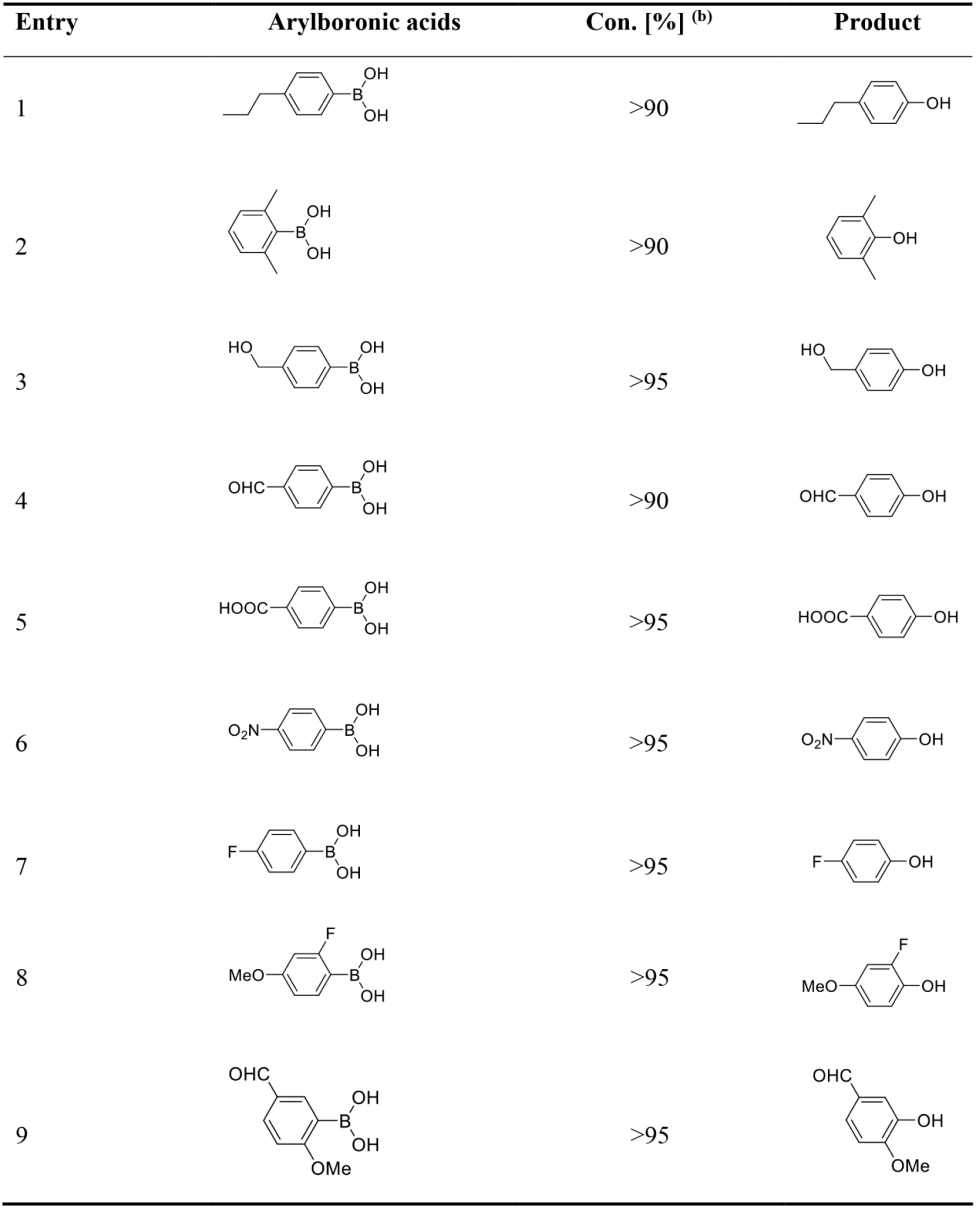
Visible-Light-Induced Aerobic Oxidative Hydroxylation of Aryl Boronic Acids^a^ ^b^

a The photocatalyst was evaluated under air and irradiation with LED light (λ = 450 nm) for 24 h. PDha-*co*-(APTMA-*co*-Ru) has a total concentration of 0.5 mg/mL, and arylboronic acids has a concentration of 0.01 mM.

b The solution was analyzed directly by ^1^H NMR spectroscopy for quantitative calculation of conversion. Hexamethyldisiloxane (HMDSO) or pyrazine was used as an internal standard.

Substrates bearing electron-withdrawing substituents, such as fluoro, nitro, and carboxylic groups, also underwent smooth conversion, providing the corresponding phenols (entries 5–7). Polar arylboronic acids were converted more efficiently than their hydrophobic counterparts, such as propyl- or dimethyl-substituted derivatives. Notably, electron-rich phenols, typically difficult to access via nucleophilic substitution of aryl halides, were readily obtained by using this photocatalytic approach. The method thus demonstrates broad functional group compatibility and robust catalytic performance across the electronic classes. Importantly, arylboronic acids bearing multiple functional groups, including combinations of electron-donating, electron-withdrawing, or sensitive functionalities, such as aldehydes, also yielded phenol products with high conversion (entries 8 and 9), highlighting the robustness and broad functional group compatibility of this catalytic system.

### Self-Assembly Pathway to Hydroxylation Mechanism with PDha-*co*-(APTMA-*co*-Ru)

The photocatalytic hydroxylation of arylboronic acids mediated by PDha-*co*-(APTMA-*co*-Ru) proceeds through a cooperative mechanism in which substrate binding triggers polymer self-assembly and enables photoredox catalysis. Hereby, under mildly basic conditions (pH 8.5–9, adjusted with *i*PrNEt_2_), deprotonation of PDha carboxyl groups increases electrostatic repulsion, extending the polymer chains and exposing substrate-binding sites. At the same time, cationic APTMA units may interact electrostatically with the polar functionalities of the arylboronic acids. Dual hydrogen bonding between boronic acid hydroxyl groups and PDha carboxylates further stabilizes the substrate–polymer complex and drives the formation of spherical nanoaggregates (Scheme S1; see also the Self-Assembly Behavior section), promoting substrate preorganization for the photoredox cycle. To support this mechanism, we performed DFT calculations on representative polymer fragments and complementary experimental spectroscopy studies on PDha-*co*-(APTMA-*co*-Ru).

The DFT results (B3LYP+D3­(BJ)/def2-SVP/CPCM­(water)) identified dual hydrogen bonding as the most energetically favorable interaction across relevant protonation states (Figure 4A, Tables S3 and S5), highlighting the robustness of this binding mode. Furthermore, 4-methoxyphenol, the product of 4-MPBA hydroxylation, is also capable of engaging in hydrogen bonding with the polymer, albeit with markedly weaker interactions with one hydrogen bond between the oxygen of the carboxyl group of the polymer and the OH group of 4-methoxyphenol (Table S6).

**Figure 4:**
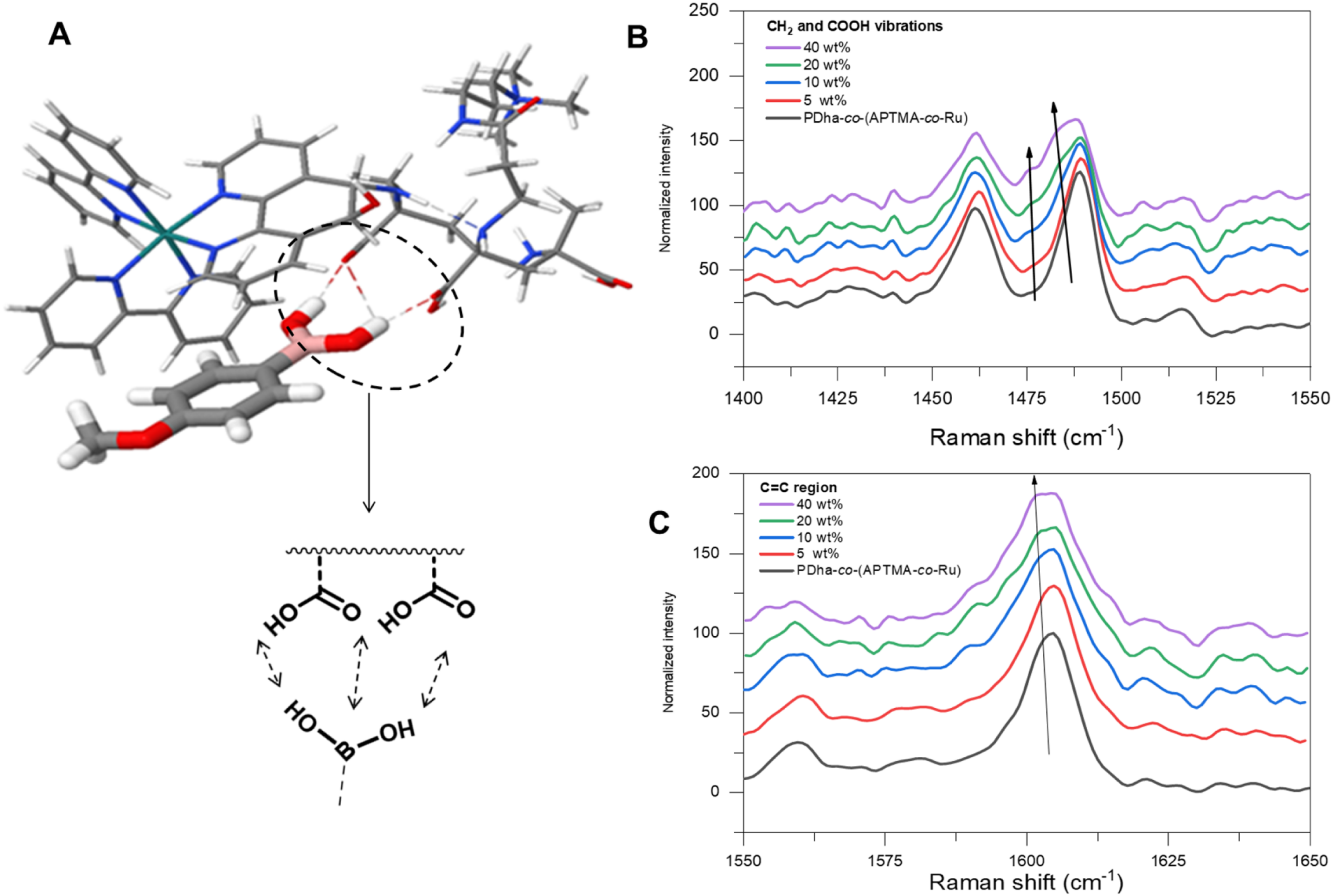
(A) Interaction of 4-MPBA with carboxyl functional groups of PDha-*co*-(APTMA-*co*-Ru) [the complex was optimized at the B3LYP+D3­(BJ)/def2-SVP/CPCM­(water) level]. (B and C) Raman spectra (C=C, −CH_2_, and COOH region) of PDha-*co*-(APTMA-*co*-Ru) (black) and PDha-*co*-(APTMA-*co*-Ru)/4-MPBA with different 4-MPBA content (red: 5 wt %, blue: 10 wt %, green: 20 wt %, and purple: 40 wt %) obtained during passive dehydration of the samples.

To experimentally support the above hypothesis regarding hydrogen-bond formation between PDha-*co*-(APTMA-*co*-Ru) and 4-MBPA, we performed Raman spectroscopy measurements at varying 4-MBPA concentrations. Raman spectroscopy is well-suited for probing intermolecular interactions such as hydrogen bonding, as Raman bands of water are weak, and subtle changes in the polarization of functional groups upon interaction often manifest as shifts in peak positions. Because the PDha backbone contains a high density of carboxylic acid groups, hydrogen bonding is expected to induce shifts of the corresponding Raman bands to lower wavenumbers.^61,62^ All measurements were conducted in a dry state. Prior to analysis, PDha-*co*-(APTMA-*co*-Ru) and 4-MBPA were mixed in solution at defined ratios and allowed to interact for 15 min. Subsequently, 25 μL of each mixture was deposited onto a clean silicon chip. Figure 4B–C and Figure S21 show the resulting Raman spectra of the dried polymer–substrate mixtures across the tested ratios. Each spectrum was accumulated over approximately 1 h of irradiation.

Overall, the spectral profiles remain largely unchanged as the 4-MBPA concentration increases. The bands at ∼1040 and 1600 cm^–1^ correspond to the ring-breathing and C=C stretching vibrations of the bipyridyl ligands, respectively, which appear prominently due to the high Raman cross-section of aromatic systems. Peaks at 1274 cm^–1^ (νC–O), 1320 cm^–1^ (δC–O–H), and 1490 cm^–1^ (ν_as_(COOH)) originate from the carboxyl-rich PDha backbone and represent the modes most sensitive to hydrogen bonding. The feature at 1460 cm^–1^ is assigned to CH_2_ scissoring in the polymer. With increasing 4-MBPA content, the characteristic ring-breathing vibration of the phenylboronic acid at ∼800 cm^–1^ becomes evident, while other 4-MBPA modes remain comparatively weak and do not significantly influence the spectra.^63,64^

As the concentration of 4-MBPA increases, most Raman bands exhibit noticeable broadening, likely reflecting a conformational transition of the polymer from an extended coil to a more compact globular structure. The ring-breathing mode of the bipyridyl ligand remains essentially unchanged; however, increasing 4-MBPA content leads to the appearance of an additional peak in the C=C stretching region at ∼1600 cm^–1^. This feature cannot be assigned to the intrinsic C=C stretching vibration of 4-MBPA, which appears at 1612 cm^–1^, and is therefore attributed to changes in the local environment of the bipyridyl rings—most plausibly a more hydrophobic microenvironment arising during aggregation. A similar effect is observed for the ν_as_(COOH) band at 1490 cm^–1^, where two new shoulders appear in the green and purple spectra at ∼1480 and ∼1475 cm^–1^. Since no Raman-active vibrations of 4-MBPA occur in this region, these additional features must originate from interactions involving the carboxylic acid groups. Strong hydrogen bonding is expected to manifest through both peak broadening and shifts to lower wavenumbers, reflecting weakened C–O and O–H bonds within the carboxyl moieties. The simultaneous emergence of a broadened shoulder at 1480 cm^–1^ and a distinct new peak at 1475 cm^–1^ therefore provides strong evidence for pronounced hydrogen bonding between PDha units and, most likely, 4-MBPA. Consistent shifts and broadening are also observed at 1274 and 1320 cm^–1^, further demonstrating that these spectral changes represent a systematic trend rather than isolated events. Together, these observations support the conclusion that hydrogen bonding plays a central role in driving polymer–substrate complexation and the subsequent formation of polymeric colloids.

Time-resolved emission spectroscopy is a powerful method to evaluate polymer hydrodynamics because it reports on the rotational diffusion of an emissive probe and thus on polymer motion and conformation. Ru­(II) polypyridyl complexes, with their long lifetimes and polarized emission, are therefore well suited to monitor the relatively slow rotational dynamics of polymer chains.^65,66^ Building on this, we followed the time-resolved emission of PDha-*co*-(APTMA-*co*-Ru) during the 4-MPBA-induced colloidal assembly to gain mechanistic insight into the hydroxylation process (Figure 5A and Figure S22).

**Figure 5:**
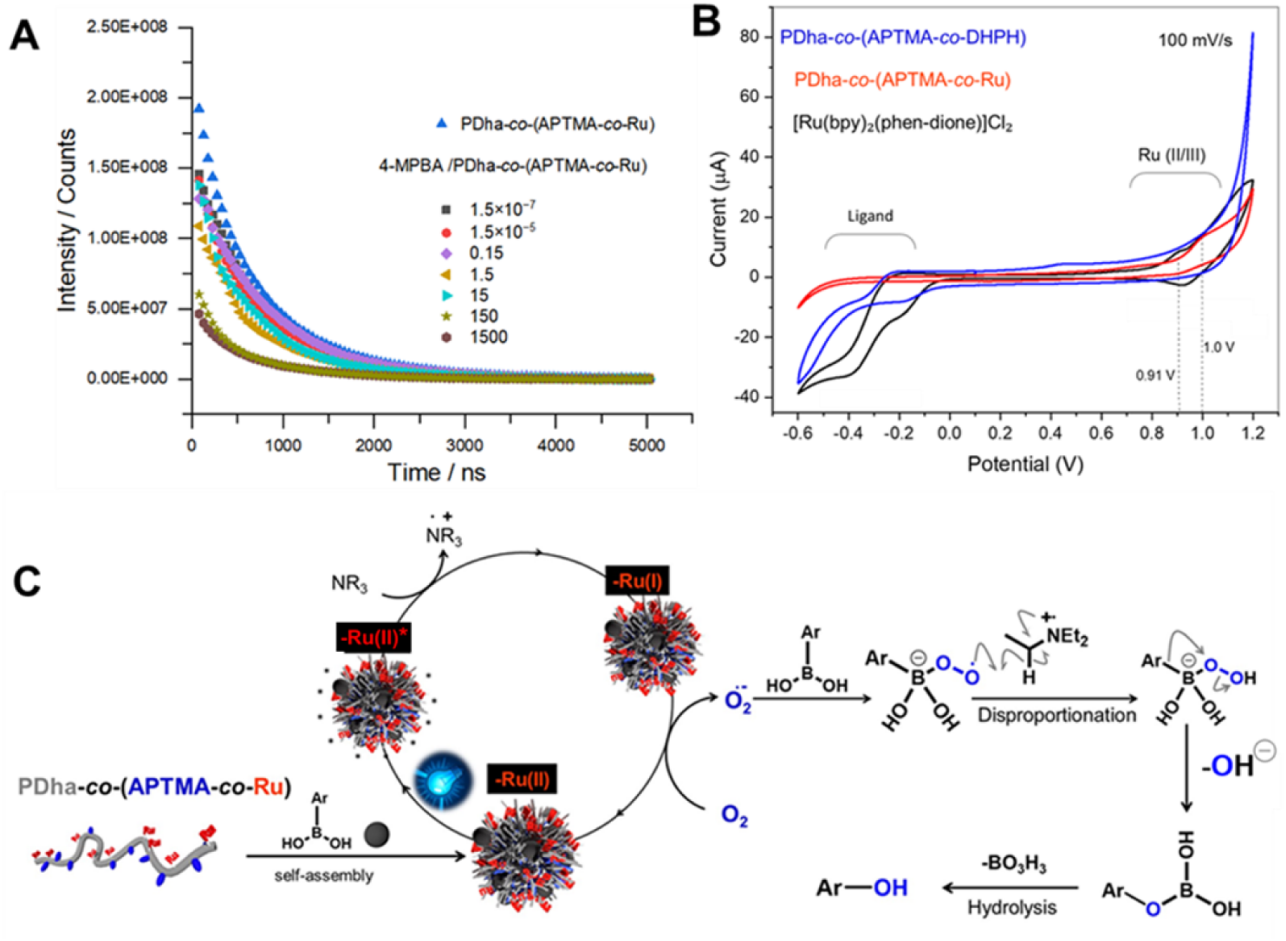
(A) Emission decay (excitation wavelength λ_ex_ = 450 nm) of aqueous PDha-*co*-(APTMA-*co*-Ru) (6.6 μg/mL) in the absence and presence of 4-MPBA at different wt/wt ratios, together with biexponential fits. (B) Cyclic voltammogram of an aqueous solution of [Ru­(bpy)_2_(phen-dione)]­Cl_2_, PDha-*co*-(APTMA-*co*-Ru), and PDha-*co*-(APTMA-*co*-DHPH)-KCl on SPCE; scan rate: 100 mV·s^–1^; potential range: −0.6 to +1.2 V vs Ag/AgCl. (C) Proposed mechanism for the formation of photocatalytically active colloidal nanoaggregates from PDha-*co*-(APTMA-*co*-Ru) and arylboronic acids, along with the subsequent oxidative hydroxylation to yield the corresponding phenol products.

Figure S22 shows the time-resolved emission spectra of PDha-*co*-(APTMA-*co*-Ru) in water, recorded in the absence and presence of increasing amounts of 4-MPBA. Upon the addition of 4-MPBA, the overall emission intensity decreases, indicating efficient quenching and providing a first spectroscopic indication of binding between PDha-*co*-(APTMA-*co*-Ru) and 4-MPBA, in agreement with our DFT and Raman data. Notably, the emission maximum of PDha-*co*-(APTMA-*co*-Ru) remains essentially unchanged upon the addition of 1500 wt/wt (as the mass ratio) of 4-MPBA, showing that the emissive MLCT state is preserved.

Emission decay parameters for PDha-*co*-(APTMA-*co*-Ru) were also determined in the absence and presence of increasing amounts of 4-MPBA (Figure 5A), and the results are listed in Table S7. No measurable emission was observed in *N,N*-dimethylacetamide (DMAc) because the PDha backbone is insoluble in this solvent, whereas well-defined decays were obtained in water, confirming that the emission arises from Ru centers whose excited-state properties are controlled by the conformation and microenvironment of the PDha chains. Emission decay kinetics shown in Figure 5A were analyzed using a biexponential decay model. PDha-*co*-(APTMA-*co*-Ru) in water exhibited a short lifetime of approximately 244 ns, contributing ∼33% of the total amplitude, and a long lifetime of about 814 ns, contributing ∼66%.

The short component is assigned to Ru centers in more solvated, exposed environments, consistent with lifetimes reported for related Ru­(II) polypyridyl complexes in an aerated aqueous solution. The second, long component is therefore attributed to Ru centers located in protected, aggregated microenvironments created by the polymer (e.g., collapsed domains or colloidal assemblies), analogous to the behavior described for Ru­(II) complexes embedded in polymer matrices, micelles, vesicles, or lipid bilayers.^65,67,68^

For all mixing ratios of PDha-*co*-(APTMA-*co*-Ru) and 4-MPBA, the luminescence decay kinetics (Figure 5A) could be fitted with the same biexponential model, keeping the lifetimes *t*1 and *t*2 fixed (set as global parameters) and allowing only the amplitudes to vary (Table S7). The amplitude changes, therefore, reflect a redistribution of Ru centers between the two microenvironments defined by *t*1 and *t*2. Upon the addition of the first small amount of 4-MPBA (4-MPBA/PDha-*co*-(APTMA-*co*-Ru) = 1.5 × 10^–7^ wt/wt), the relative amplitude of the short component decreases from ∼0.33 to 0.18, while that of the long component increases from ∼0.67 to 0.82. This indicates that the fraction of solvated, exposed Ru centers (associated with *t*1) decreases, and more Ru is transferred into protected, aggregated domains (associated with *t*1). In neutral solution, the PDha side chains are largely deprotonated; upon the addition of 4-MPBA, −B­(OH)_2_ functional groups can form hydrogen-bonded boronate–carboxylate contacts with these groups, which partially “locks” the COOH/COO^–^ functions and drives the polymer into a more compact, aggregated conformation. In this rearranged state, Ru centers are increasingly embedded in PDha-rich domains, where they are better shielded from solvent and external quenchers. At higher 4-MPBA contents, the amplitude of *t*1 increases again, while that of *t*2 decreases. For example, A1 rises from ∼0.18 at 1.5 × 10^–7^ (w/w) 4-MPBA to ∼0.42–0.60 at 1.5–1.5 × 10^2^, whereas A2 falls from ∼0.82 to ∼0.40–0.58. This progression is consistent with the formation of larger, roughly spherical colloidal aggregates in which an increasing fraction of Ru centers are located at or near the particle surface. Such spherical aggregates present more surface area to the solvent, so surface-bound Ru sites experience greater solvent exposure and more frequent encounters with neighboring chromophores, leading to enhanced quenching; subsequently, a reduced contribution from the long-lived, well-protected population to the overall luminescence kinetics is observed. The evolution of the relative amplitudes with 4-MPBA concentration suggests that colloidal aggregation and polymer self-assembly modulate the local electronic environment of the Ru centers and, consequently, their excited-state dynamics.

To probe the electrochemical behavior of the polymeric photocatalyst, we performed stepwise cyclic voltammetry (CV) measurements across different stages of polymer functionalization. By comparing the CV signatures of a model Ru complex, PDha-*co*-(APTMA-*co*-DHPH), and PDha-*co*-(APTMA-*co*-Ru), we could unambiguously assign the observed redox features to the covalently incorporated Ru centers (Figure 5B and Figure S23).

As a structural model of the polymer-anchored ligand, 1,10-phenanthroline-5,6-dione (phen-dione) was first synthesized and subsequently coordinated to Ru­(II) to form the model complex [Ru­(bpy)_2_(phen-dione)]­Cl_2_, thereby representing the coordination environment of the polymer-bound Ru sites (see details in the Supporting Information). We compared its properties with those of [Ru­(bpy)_2_Cl_2_], PDha-*co*-(APTMA-*co*-DHPH), and PDha-*co*-(APTMA-*co*-Ru). We observed a stepwise appearance and disappearance of oxidation and reduction peaks associated with both the coordination sites and the Ru centers within our polymer. Notably, the polymer-bound Ru complex exhibited an oxidation potential of *E*_ox_ ≈ 1.0 V vs. Ag/AgCl, which is comparable to that of [Ru­(bpy)_2_Cl_2_] (1.2 V) and the model complex (0.9 V), indicating that incorporation into the polymeric scaffold only subtly alters the electronic environment of the Ru centers, while potentially impacting their catalytic behavior. However, the reduction wave of the Ru complex could not be resolved due to the onset of water reduction beyond −0.6 V vs NHE, which imposes limitations on electrochemical characterization under aqueous conditions. According to previous observations,^60,69^ photoexcitation of the Ru­(II) moiety, followed by reductive quenching by NEt_3_, generates a Ru­(I) intermediate that reduces molecular oxygen to the superoxide radical anion (O_2_˙^–^), as supported by redox potentials and prior mechanistic reports (Figure 5C).^60^ This reactive oxygen species initiates hydroxylation via nucleophilic attack at the boron center, generating a peroxide radical intermediate that undergoes hydrogen abstraction, rearrangement, and subsequent hydrolytic conversion to the phenol product. The polymer thus serves not only as a catalytic scaffold but also as a dynamic microenvironment that stabilizes reactive intermediates and lowers activation barriers, likely through the compartmentalization and preorganization of reactants. This highlights a synergistic interplay between supramolecular self-assembly and redox catalysis, enabling efficient photocatalysis.

## Conclusion

This study establishes a proof-of-concept for integrating photocatalytically active species into a soft, adaptive polymer matrix. To create such a platform, we synthesized a water-soluble PDha-based copolymer functionalized with Ru polypyridyl centers. In aqueous media containing hydrophobic reactants, these copolymers spontaneously form spherical nano-objects that act as catalytic nanoreactors, as supported by spectroscopic characterization and time-resolved measurements.

These nanostructures exhibit high reactivity in the photocatalytic hydroxylation of arylboronic acids through a cooperative mechanism, in which self-assembly and photoredox catalysis act synergistically. Raman spectroscopy and DFT calculations indicate specific hydrogen-bonding interactions between carboxylic acid groups on the PDha backbone and boronic acid moieties, which facilitate aggregate formation and organization in water. Time-resolved emission analyses further show a redistribution of Ru­(II) centers from solvated to protected microenvironments upon 4-MPBA binding, followed by the emergence of colloidal aggregates with Ru sites at or near the particle surface, consistent with an assembly-controlled modulation of the Ru excited-state dynamics. The polymeric nanoreactors enhance catalytic performance by improving the solubility and dispersion of both catalysts and substrates and may also promote efficient excited-state energy and/or electron transfer within the aggregates. Their efficacy is demonstrated by the light-driven conversion of arylboronic acids to phenols with high conversion while tolerating electron-withdrawing substituents and sensitive functional groups. Overall, this photocatalytically active copolymeric system enables mild aqueous reaction conditions and provides a route for the selective generation of electron-rich phenols. These results highlight the potential of rationally designed polymer–catalyst conjugates as tunable nanoreactors for aqueous photoredox catalysis. Future efforts will focus on optimizing copolymer architecture to enhance colloidal stability, sharpen control over Ru microenvironments, expand the substrate scope, and broaden the range of photocatalytic transformations accessible through these adaptive assemblies.

## Supplementary Material



## Notes

Detailed synthetic procedures, structural analysis, and characterizations of the prepared polymer and phenols; computational details and results; and additional experimental data, including TGA, DLS, TEM, Raman spectra, time-resolved emission spectra, and cyclic voltammetry (PDF)
