# Supplementary material for: Polyelectrolyte Copolymer Nanoreactors: From Colloidal Assembly to Photoredox Activity in Water
Source: ACS Appl Mater Interfaces. 2026 Mar 16;18(13):19622–34. doi: 10.1021/acsami.6c00473 (PMC13067237; doi:10.1021/acsami.6c00473)
Supplement: Supplementary file 1 [file am6c00473_si_001.pdf]

Supporting Information

Polyelectrolyte Copolymer Nanoreactors: From Colloidal Assembly to Photoredox Activity in Water

Afshin Nabiyan*, Mitra Esfandiari, Sergio Kogikoski Jr, Ilko Bald, Evgenii Titov, Michael U. Kumke, Nora Kulak, Helmut Schlaad

Institute of Chemistry, University of Potsdam, Karl-Liebknecht-Str. 24-25, 14476 Potsdam, Germany

Afshin.nabiyan@uni-potsdam.de

Synthesis of M*t*BAMA

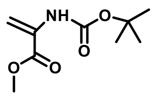

Boc-Ser-OMe (10.0 g, 45.6 mmol, 1.0 equiv) was dissolved in dichloromethane (200 mL) in a reaction flask, followed by the dropwise addition of methanesulfonyl chloride (MsCl, 6 mL, 77.5 mmol, 1.7 equiv) while stirring. The reaction mixture was cooled to 0 °C in an ice bath, and triethylamine (23 mL, 165.9 mmol, 3.6 equiv) was slowly added dropwise over 10 min, ensuring the temperature remained at 0 °C. The mixture was stirred at this temperature for 1 h, then allowed to warm to room temperature and stirred for additional 2 h. The reaction mixture was transferred to a separatory funnel and washed with 200 mL of 1% aqueous potassium bisulfate solution. The organic phase was separated and dried over anhydrous sodium or magnesium sulfate, then filtered, and the solvent was removed under reduced pressure using a rotary evaporator. The crude product was purified by column chromatography on silica gel, using a mixture of ethyl acetate and *n*-hexane (1:4 v/v) as the mobile phase. Product fractions were collected, combined, and the solvent was removed under reduced pressure, resulting in the final product as a colorless oil (8.6 g, 94% yield). ^{1}H NMR (300 MHz, $CDCl_3$): δ 7.01 (s, 1 H, NH), 6.10, 5.73 (s, 1 H, $=CH_2$), 3.82 (s, 3 H, $-OCH_3$), 1.45 (s, 9 H, $-C(CH_3)_3$) ppm.

Synthesis of PM*t*BAMA

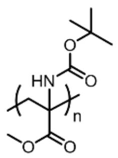

N-(2-Methylpropyl)-*N*-(1-diethylphosphono-2,2-dimethylpropyl)-*O*-(2-carboxylprop-2-yl)-hydroxylamine (MAMA-SG1 or BlocBuilder®) and *N*-(2-methyl-propyl)-*N*-(1-diethyl-phosphono-2,2-dimethylpropyl)-*N*-oxyl) (SG1) were synthesized based on previous reports in literature.[1–3]. M*t*BAMA, MAMA-SG1, and SG1 (molar ratio 40:1:0.65) were dissolved in 1,4-dioxane to afford a 66 wt% solution. The reaction mixture was subjected to four freeze–pump–thaw cycles to rigorously remove dissolved gases and ensure an oxygen-free environment. Subsequently, the solution was stirred at 80 °C under an argon atmosphere for 15 min. Upon completion, the crude polymer was precipitated by slow addition into cold n-hexane. The precipitate was collected by filtration, washed thrice with hexane to remove residual impurities, and dried under reduced pressure, yielding a white powder. ^{1}H NMR (300 MHz, $CDCl_3$): δ 6.00–5.00 (m, NH), 4.0–3.3 (m, $-OCH_3$), 3.3–2.0 (m, $-CH_2-$), 1.7–1.1 (m, $-C(CH_3)_3$) ppm. SEC (THF, PMMA calibration): M_n 70.0 kg/mol, $Đ$ 1.5.

Deprotection of PM*t*BAMA

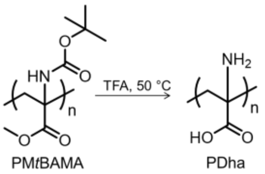

PM*t*BAMA was dissolved in trifluoroacetic acid (TFA) at a concentration of approximately 40 equiv per monomer unit and heated to 50 °C. The reaction was maintained at this temperature for 1 h. Upon completion, the mixture was poured into cold methanol to precipitate the product. The precipitate was collected by filtration and washed multiple times with cold methanol to remove residual TFA, followed by washes with diethyl ether to eliminate remaining impurities. The product was then dried under vacuum, affording PDha as a colorless powder in 60% yield. ^{1}H NMR (300 MHz, D_2O + NaOD): δ 3.3 (s, $-OCH_3$), 2.8–2.0 (m, $-CH_2-$) ppm. Degree of methyl ester hydrolysis ~93%.

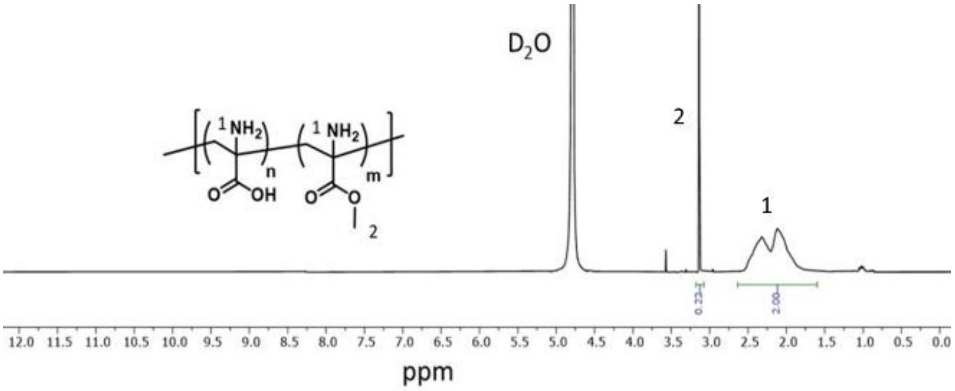

Figure S1: ^{1}H NMR (300 MHz, D_2O + NaOD) spectrum of PDha obtained by deprotection of PM*t*BAMA with TFA.

Modification of PDha with APTMA

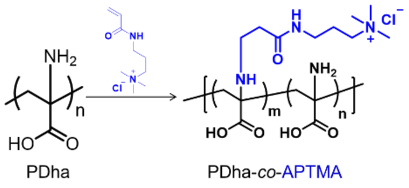

PDha (100 mg) and APTMA (5 equivalents per monomer unit) were separately dissolved in water (5 mL each, pH 13 adjusted with KOH). The clear solutions were combined and stirred at 60 °C in an oil bath for 24 h. The reaction was quenched by the gradual addition of 0.5 M aqueous HCl until the solution reached pH 7. The crude product was then dialyzed against deionized water (MWCO 3.5 kDa) for 3 d, followed by freeze-drying to yield PDHa-APTMA as a light orange powder.

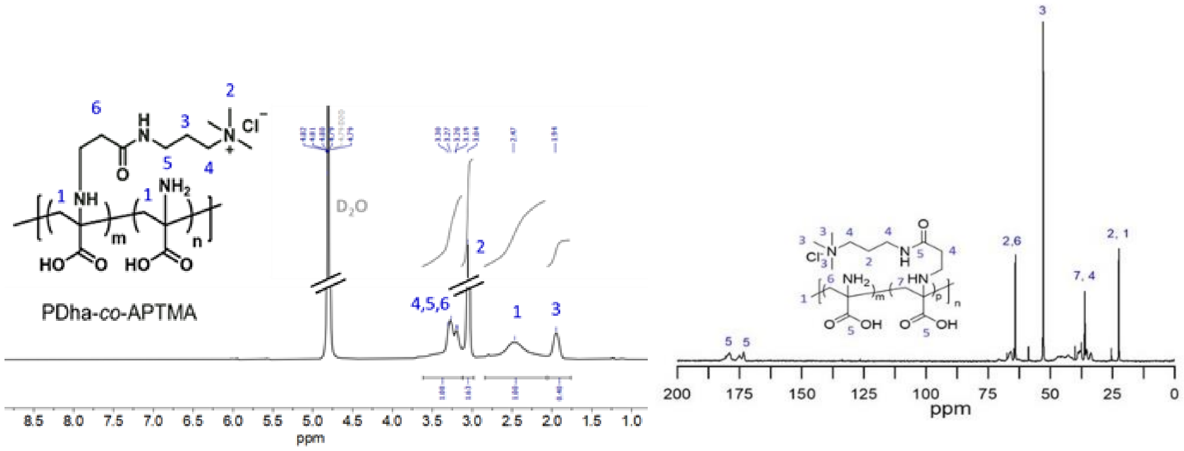

Figure S2: ^{1}H NMR (300 MHz) (left) and ^{13}C NMR (75 MHz) (right) spectra of PDha-*co*-APTMA in D_2O/NaOD (pH 8-9).

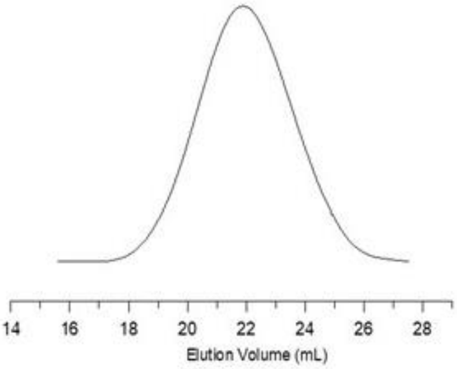

Figure S3: SEC-RI trace (eluent: water with 0.3% TFA/0.1 M NaCl, pH < 2) of PDha-*co*-APTMA.

Modification of PDha-*co*-APTMA with DHPA

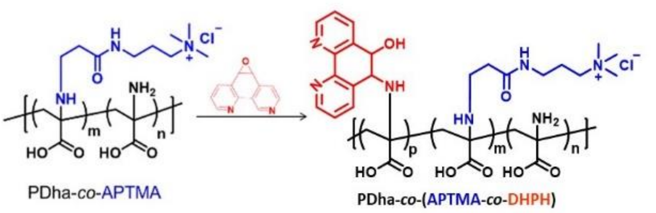

PDha-*co*-APTMA (100 mg) was dissolved in 5 mL of water adjusted to pH 13 with KOH. Separately, 1a,9b-Dihydrooxireno[2,3-f][1,10]phenanthroline (5 equiv per monomer unit) was dissolved in a 1:1 (v/v) mixture of water and DMSO (2.5 mL each) along with 0.5 mL of DBU. The solutions were combined and stirred at 65 °C under an argon atmosphere for 48 h. The reaction was quenched by adjusting the pH to 7 with 0.5 M aqueous HCl. The crude product was dialyzed against deionized water using a membrane with a 3.5 kDa molecular weight cutoff for 3 d, followed by freeze-drying to yield PDha-*co*-(APTMA-*co*-DHPA) as a yellow/golden powder.

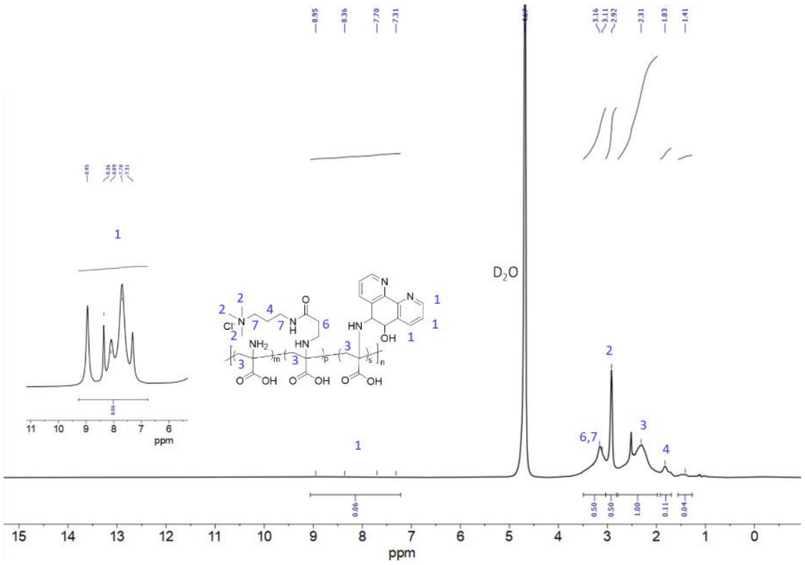

Figure S4: ^{1}H NMR (300 MHz) spectrum of PDha-*co*-(APTMA-*co*-DHPH) in D_2O

Ru attachment to PDha-*co*-(APTMA-*co*-DHPH)

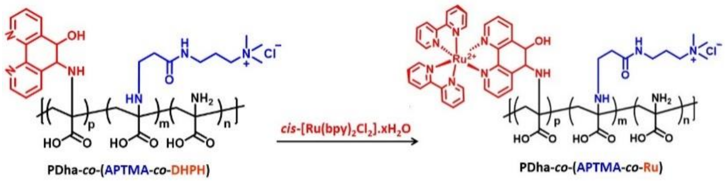

100 mg of PDha-*co*-(APTMA-*co*-DHPA) was dissolved in water. Separately, 100 mg of *cis*-bis(2,2′-bipyridine)dichloro-ruthenium (II) was dissolved in 4 mL of a 1:1 (v/v) ethanol/water mixture and degassed with argon for 30 min. The two solutions were combined in a sealed 20 mL microwave vial and heated at 120 °C with stirring for 12 h to promote coupling. After cooling to room temperature, the crude product was dialyzed against deionized water using a 3.5 kDa molecular weight cutoff membrane for 3 d. The purified PDha-*co*-(APTMA-*co*-Ru) was obtained as a dark orange to red powder after freeze-drying.

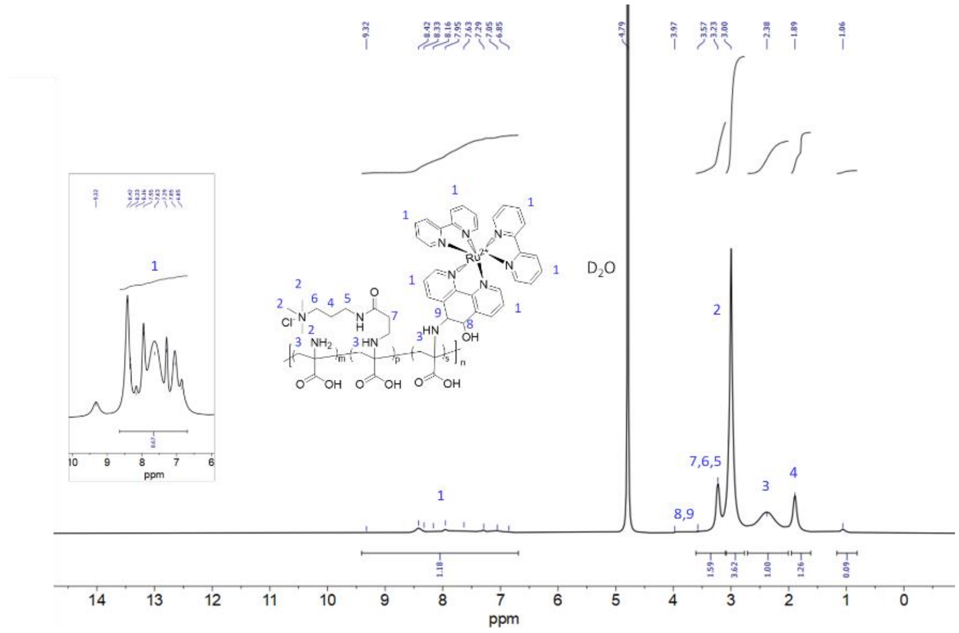

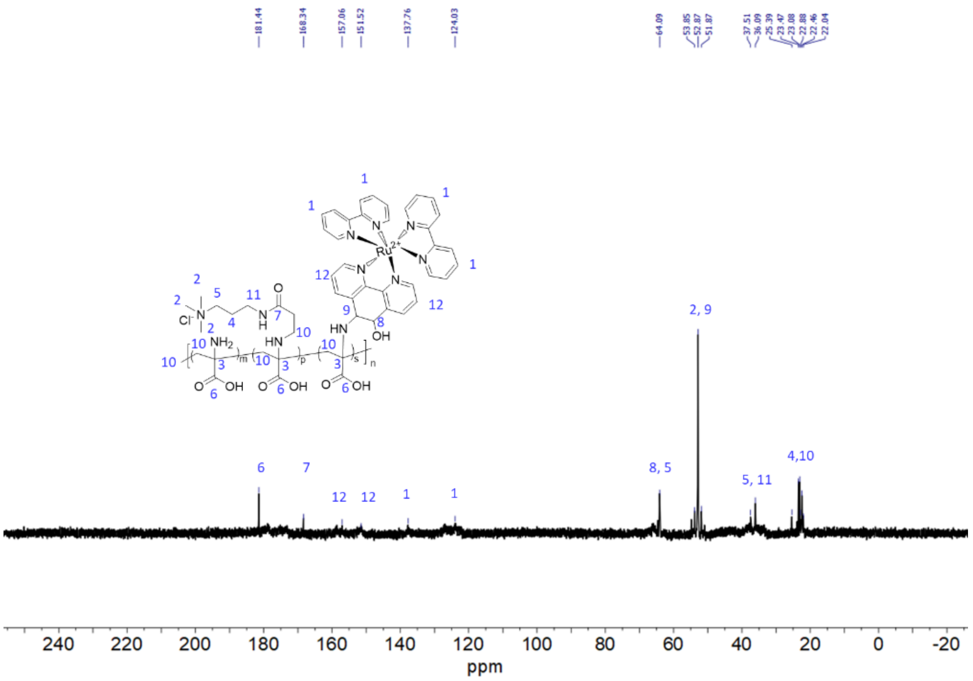

Figure S5: ^{1}H NMR (300 MHz) (top) and ^{13}C NMR (75 MHz) (bottom) spectra of PDha-*co*-(APTMA-*co*-Ru) in D_2O.

Synthesis of $[Ru(bpy)_2(phen\text{-}dione)]Cl_2$ as a model complex

$[Ru(bpy)_2(phen\text{-}dione)]Cl_2$ was synthesized with slight modifications according to literature procedures.[4–6] A mixture of $[Ru(bpy)_2Cl_2]$ (100 mg, 0.21 mmol) and 1,10-phenanthroline-5,6-dione (phen-dione) (43.3 mg, 0.21 mmol) was dissolved in 30 mL of dry ethanol and heated to reflux under a nitrogen atmosphere for 5 h with continuous stirring. Upon completion, the reaction mixture was allowed to cool to room temperature, and the solvent volume was reduced to approximately 10 mL by rotary evaporation. Precipitation of the complex was achieved by the dropwise addition of NH_4PF_6 (84.1 mg, 0.52 mmol) dissolved in 2 mL of distilled water. The resulting orange precipitate was collected by filtration, thoroughly washed with distilled water, ethanol, and diethyl ether, and subsequently dried under vacuum.

To obtain the corresponding chloride salt, the hexafluorophosphate salt of the complex (49.6 mg, 0.054 mmol) was dissolved in 1 mL of acetone. In a separate vessel, tetrabutylammonium chloride (37 mg, 0.12 mmol) was dissolved in 1 mL of acetone and then added to the complex solution. The chloride salt precipitated immediately upon mixing. The precipitate was collected by filtration, washed with cold acetonitrile, and dried under vacuum. ^{1}H NMR (400 MHz, DMSO-d_6) δ 8.97 – 8.80 (d, *J* = 8.0 Hz, 4H), 8.59 – 8.44 (d, *J* = 8.0 Hz, 2H), 8.27 – 8.12 (m, 4H), 8.02 – 7.89 (d, *J* = 8.0 Hz, 2H), 7.86 – 7.66 (m, 6H), 7.61 – 7.47 (m, 4H).

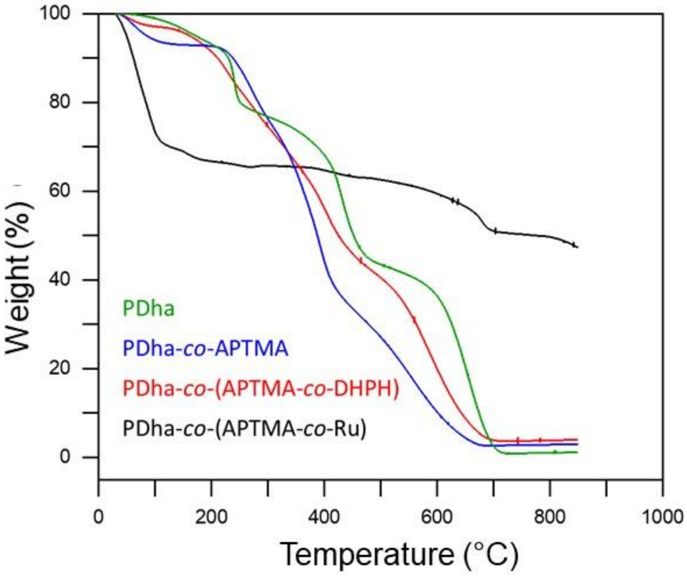

Figure S6: Thermogravimetric analysis of PDha (green) polymer, PDha-*co*-APTMA (blue), PDha-*co*-(APTMA-*co*-DHPH) (red), and PDha-*co*-(APTMA-*co*-Ru) (black) copolymers.

Computational details

Quantum chemical calculations were performed with the ORCA 6.0.1 program.[7,8] The structural models were optimized using density functional theory at the B3LYP[9] level with the D3 dispersion correction with Becke–Johnson (BJ) damping.[10] The def2-SVP basis set was used.[11] The solvent effects were modelled with conductor-like polarizable continuum model (CPCM).[12,13] The resolution of identity for the Coulomb term[14] and chain of spheres for the exchange term (RIJCOSX)[15,16] approximation (with the def2/J auxiliary basis set) was used.[17] Vertical electronic transitions to 100 lowest singlet excited states were calculated using time-dependent DFT (TD-DFT)[18] at the same B3LYP/def2-SVP/CPCM level, which was successfully used in previous calculations of Ru complexes.[19]

The broadened absorption spectra were calculated as follows:[20]

$$A(\lambda) = \sum_i f_i \exp\left(-\frac{1}{2\sigma^2}\left(\frac{1}{\lambda} - \frac{1}{\lambda_i}\right)^2\right)$$

Here, A is absorbance (in arbitrary units), λ is wavelength, f_i and λ_i are calculated oscillator strengths and excitation wavelengths of electronic transitions, respectively, and σ is a broadening parameter (chosen to be 1500 cm^{-1}). The nature of the electronic transitions was analyzed using charge density difference (CDD) plots, showing the difference between excited state and ground state electron densities, which were obtained with ORCA.

Table S1: Various ligand systems of Ru corresponding to Figure S7.

PDha-*co*-(APTMA-*co*-Ru)	**Ru** [a]	$[Ru(bipy)_2(phen\text{-}dione)]^{2+}$	$[Ru(bipy)_2Cl_2]$
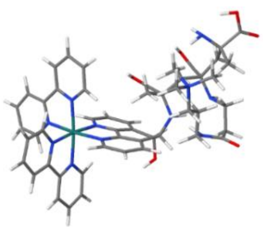	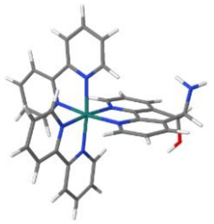	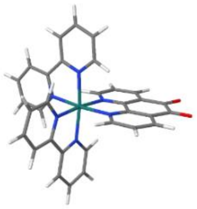	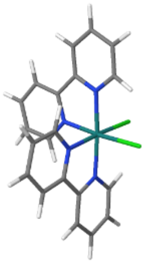

[a] Here, and in what follows, **Ru** stands for the shown Ru complex, which is part of the polymer unit.

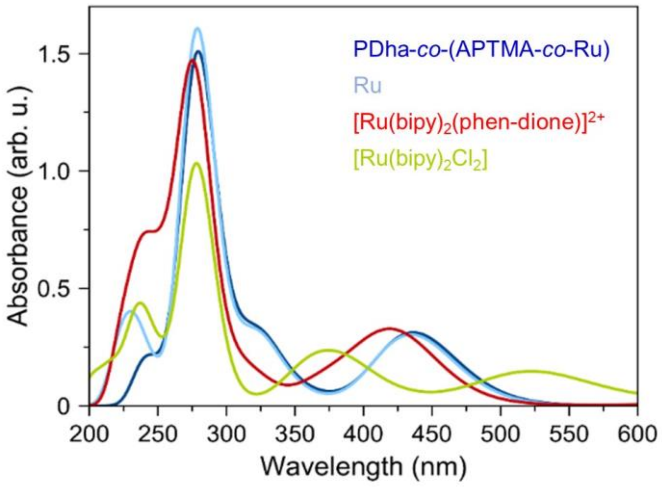

Figure S7 Calculated absorption spectra of the polymer unit and Ru complexes (see Table S1). Calculations were done with TD-B3LYP/def2-SVP using CPCM(water) for all systems but $[Ru(bipy)_2Cl_2]$, for which CPCM(acetonitrile) was used.

Table S2: Charge density difference (CDD) plots (excitation from cyan to magenta) for selected (bright) states. Transition wavelengths and oscillator strengths (in parentheses) are given.

PDha-*co*-(APTMA-*co*-Ru) repeating unit model

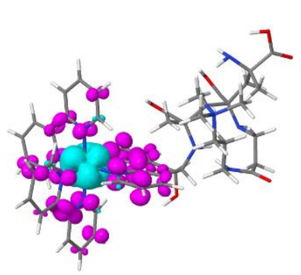

S_7, 435 nm (0.12)

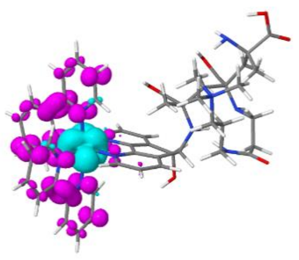

S_8, 430 nm (0.13)

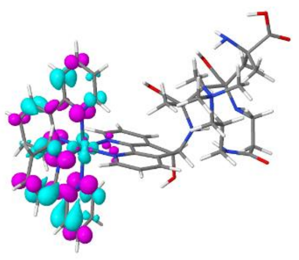

S_{55}, 277 nm (0.82)

Ru

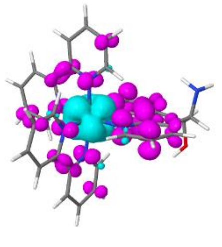

S_7, 433 nm (0.13)

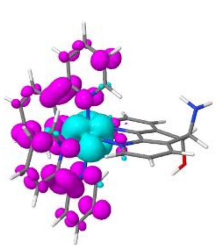

S_8, 430 nm (0.13)

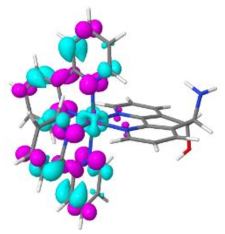

S_{43}, 276 nm (0.93)

$[Ru(bipy)_2(phen\text{-}dione)]^{2+}$

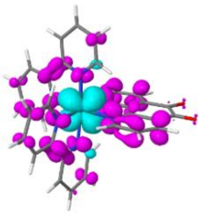

S_9, 438 nm (0.09)

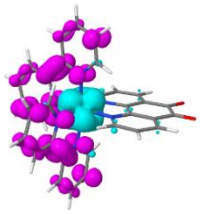

S_{12}, 417 nm (0.14)

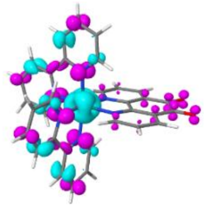

S_{47}, 276 nm (0.74)

$[Ru(bipy)_2Cl_2]$

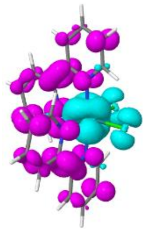

S_5, 525 nm (0.14)

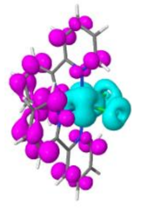

S_{11}, 394 nm (0.08)

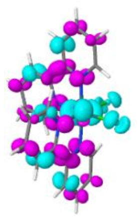

S_{37}, 277 nm (0.49)

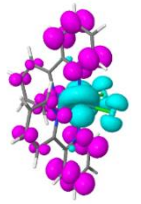

S_{18}, 358 nm (0.08)

Nanoaggregate fabrication of PDha-*co*-(APTMA-*co*-Ru) with arylboronic acids

Hybrid nanoaggregates were formed by simple mixing. PDha-*co*-(APTMA-*co*-Ru) was dissolved in deionized water at a concentration of 1.0 mg/mL. Upon addition of 4-MPBA, an initial precipitation was observed due to its poor water solubility. However, continued stirring at room temperature for 30 min led to gradual dissolution and homogeneous dispersion. This behavior suggests specific interactions between 4-MPBA and the polymer complex, promoting the formation of stable nanoaggregates in aqueous media.

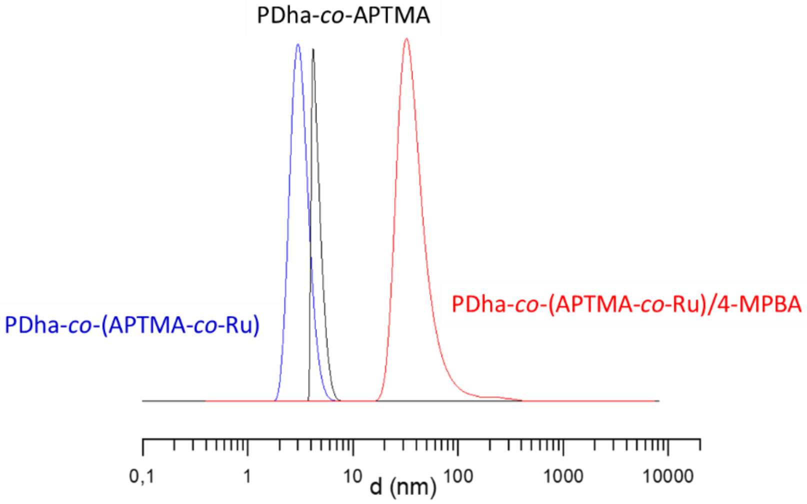

Figure S8: DLS size distributions by intensity of dilute solutions of PDha-*co*-APTMA, PDha-*co*-(APTMA-*co*-Ru) copolymer, and PDha-*co*-(APTMA-*co*-Ru)/4-MPBA hybrid nanoaggregates in water

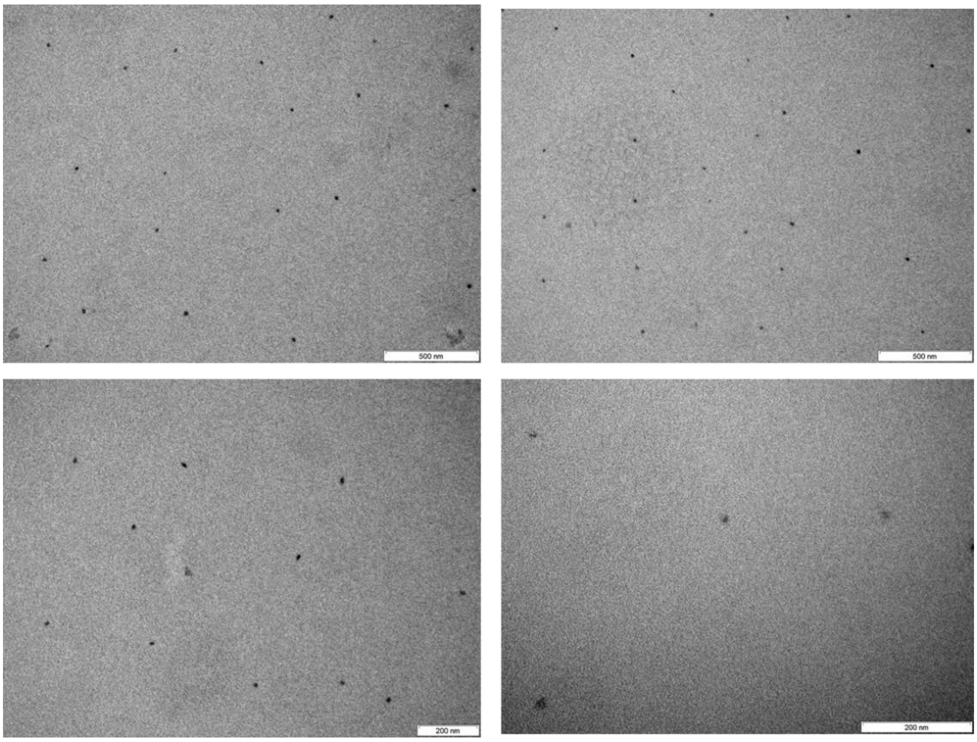

Figure S9: TEM micrographs of PDha-*co*-(APTMA-*co*-Ru)/4-MPBA with weight ratio of 1:5 (0.1 wt% polymer in water).

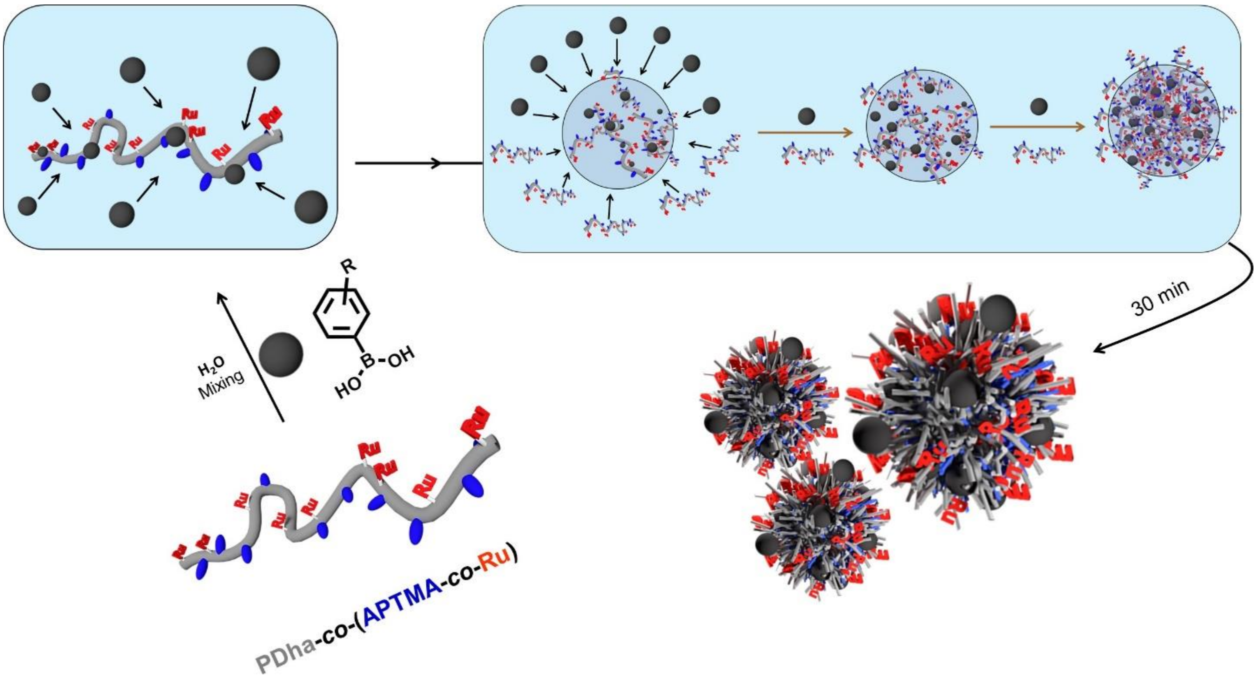

Scheme S1: Schematic illustration of substrate-induced reorganization of the copolymer and formation of colloidal nanoaggregates upon interaction with 4-MPBA.

Photocatalytic dihydroxylation of arylboronic acids

Photoreactor: Photocatalytic experiments were conducted in a custom-built 3D-printed photoreactor equipped with a ventilating fan and a blue LED light source (Opulent Americas SST-10-B-B90-Q450, λ_max = 450 ± 50 nm, 590 mW, 350 mA, 3 V).[21,22]

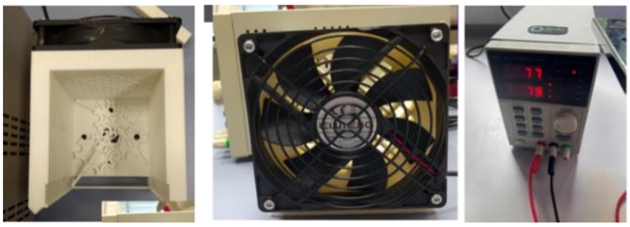

Top to side view of the photoreactor used for experiments.

Sample preparation: Typical example of photocatalytic dihydroxylation reactions of arylboronic acids were conducted in 5- or 10-mL Pyrex vials sealed with silicone septa and maintained at ambient temperature. In a standard procedure, arylboronic acid (10.0 mg) and diisopropylethylamine (50 µL) were added to a stock solution (3.0 mL, 1.0 mg/mL) of the photocatalyst in pure water. The resulting mixture was stirred for 30 min to promote the formation of nanoscale aggregate-based catalytic assemblies. Irradiation was performed using a high-power blue LED (Opulent Americas, LZ4-40B208-0000, λ_{max} = 460 ± 50 nm, 300 mW, 400 mA, 12 V) housed within a custom 3D-printed photoreactor equipped with active air cooling, as described previously. Following irradiation, the reaction mixture was extracted three times with ethyl acetate, washed with brine, and dried over anhydrous $MgSO_4$. After filtration and solvent removal under reduced pressure, the crude product was purified by flash column chromatography (silica gel, 5–20% ethyl acetate in hexanes) or preparative thin-layer chromatography to afford the corresponding phenol.

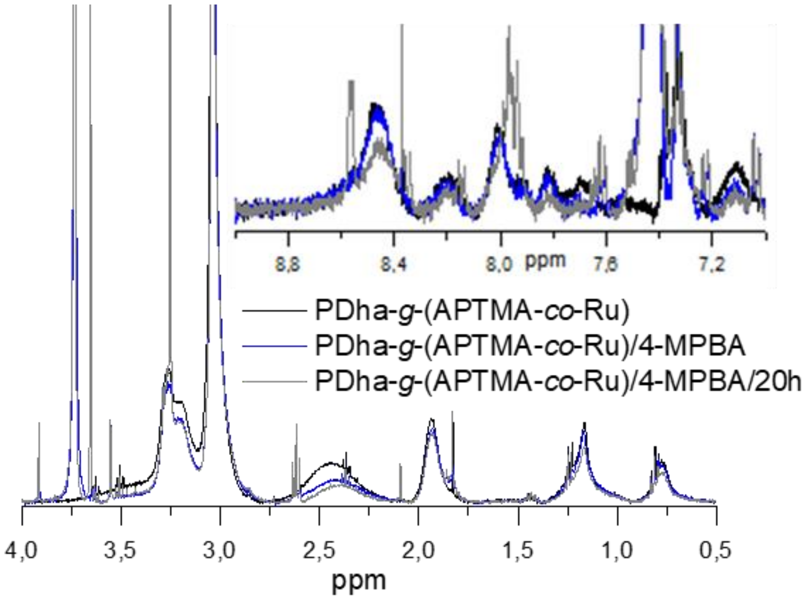

Figure S10. ^{1}H NMR spectra (400 MHz, D_2O) of the reaction mixture containing the polymer PDha-co-(APTMA-co-Ru) (0.5 mg mL^{-1}) and 4-MPBA ([4-MPBA] = 0.01 mM), recorded at different reaction times, showing the evolution of characteristic signals of the polymer backbone.

Table S3: Optimized structures of complexes of the polymer repeating unit models and 4-MPBA and their relative energies.

1

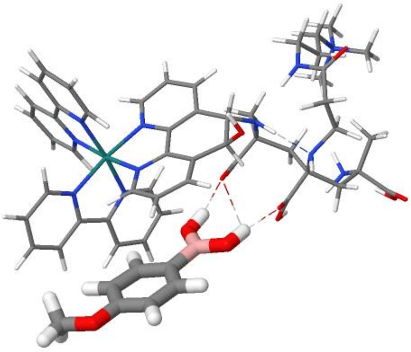

0.00 eV

2

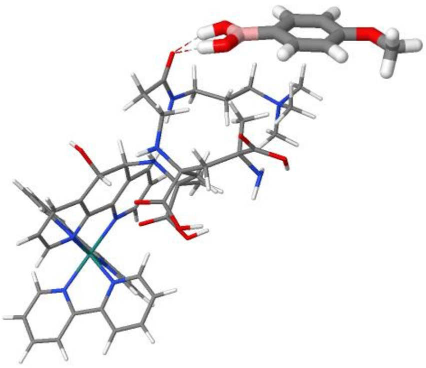

0.18 eV

3

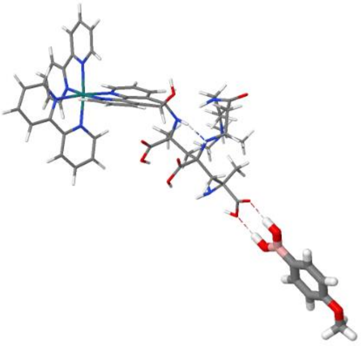

0.56 eV

4

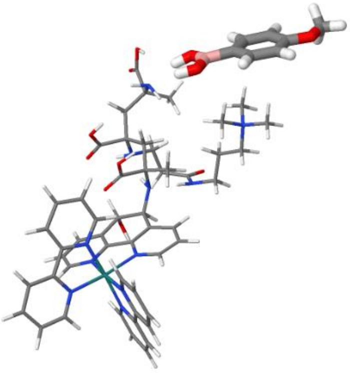

0.57 eV

5

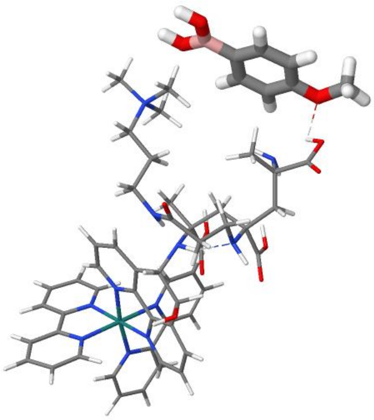

0.60 eV

Table S4: Optimized structures of complexes of the deprotonated polymer repeating unit models and 4-MPBA, and their relative energies.

1	2
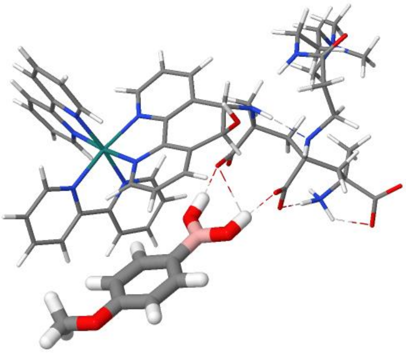	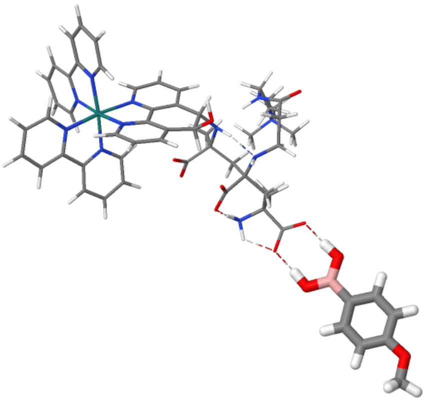
0.00 eV	0.58 eV
3	
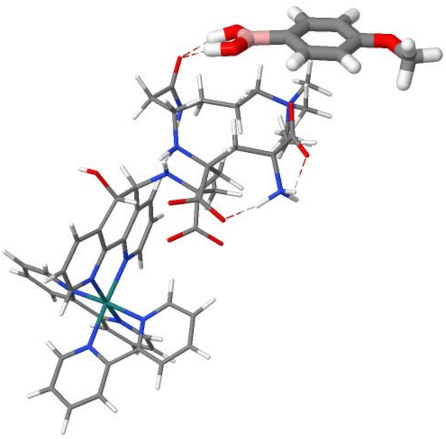	
0.60 eV	

Table S5: Complexes of 4-MPBA with polymer repeating unit models without **Ru**/APTMA.

without **Ru**	without APTMA	without **Ru** and APTMA
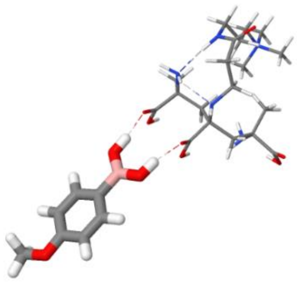	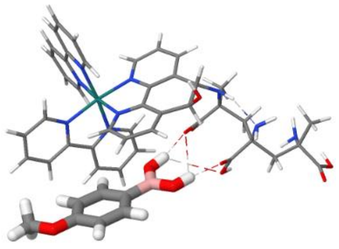	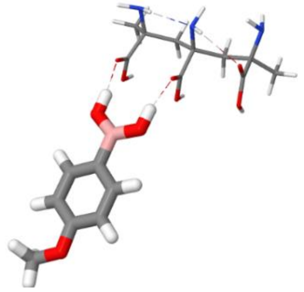

Table S6: Complexes of 4-methoxyphenol with polymer repeating unit models.

with **Ru** and APTMA	without **Ru** and APTMA
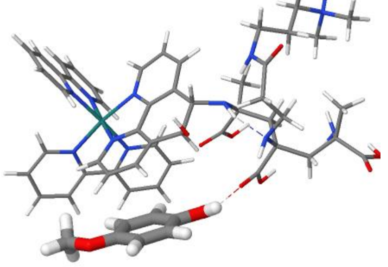	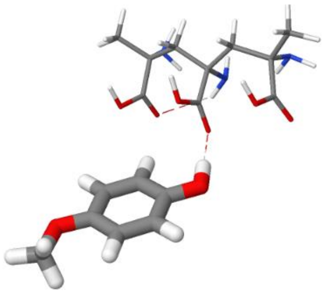

NMR and GC-MS analyses corresponding to Table 1, entry 4 and Table 2, entries 1-9, summarizing the scope of hydroxylation of arylboronic acids by PDha-*co*-(APTMA-*co*-Ru).

Table 1, entry 4: The product was isolated as a colorless to white solid by extraction with ethyl acetate and purification by column chromatography. The obtained analytical data matched the values reported for 4-methoxyphenol.[23,24] ^{1}H NMR (400 MHz, MeOD): δ 6.78 – 6.67 (m, 4H), 3.71 (s, 3H). *m/z* calculated for [M$^+$]: 124.1; found: 124.1.

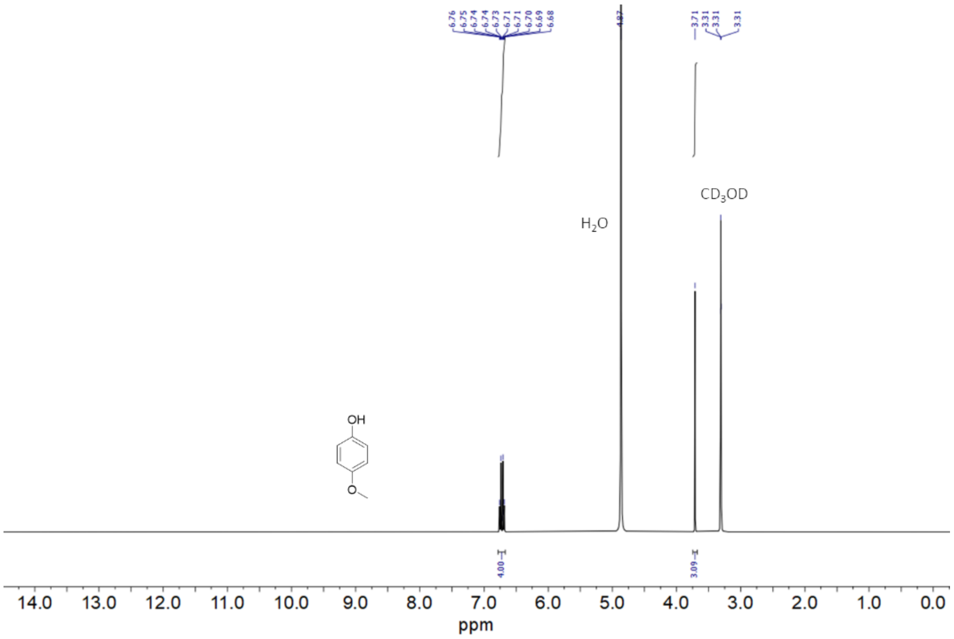

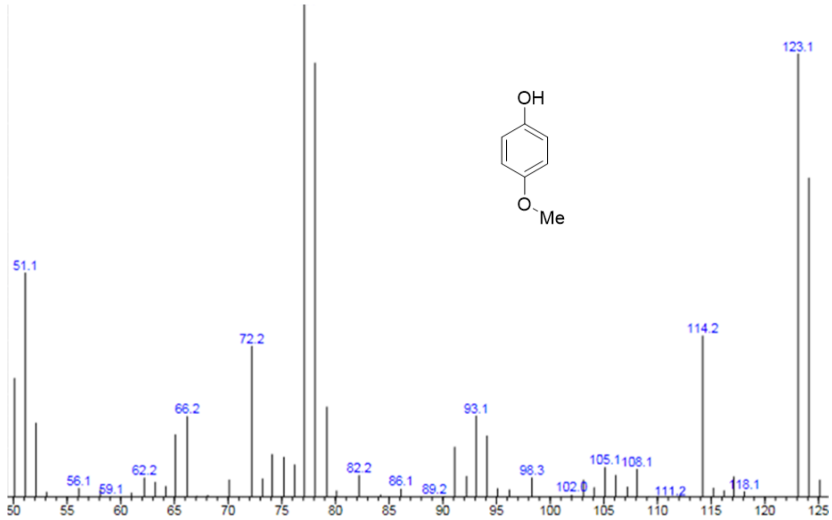

Figure S11: ^{1}H NMR (400 MHz) and mass spectra of the product from Table 1, entry 4.

Table 2, entry 1: The product was isolated as a yellow solid by extraction with ethyl acetate and purification by column chromatography. The obtained analytical data matched the values reported for 4-propylphenol.[25] ^{1}H NMR (400 MHz, MeOD) δ 7.01 – 6.93 (m, 2H), 6.71 – 6.65 (m, 2H), 2.48 (dd, *J* = 8.4, 6.7 Hz, 2H), 1.64 – 1.53 (m, 2H), 0.90 (d, *J* = 7.4 Hz, 3H). ^{13}C NMR (101 MHz, MeOD) δ 155.8, 134.2, 129.9, 115.5, 37.8, 25.6, 13.6. *m/z* calculated for [M$^+$]: 136.2; found: 136.2.

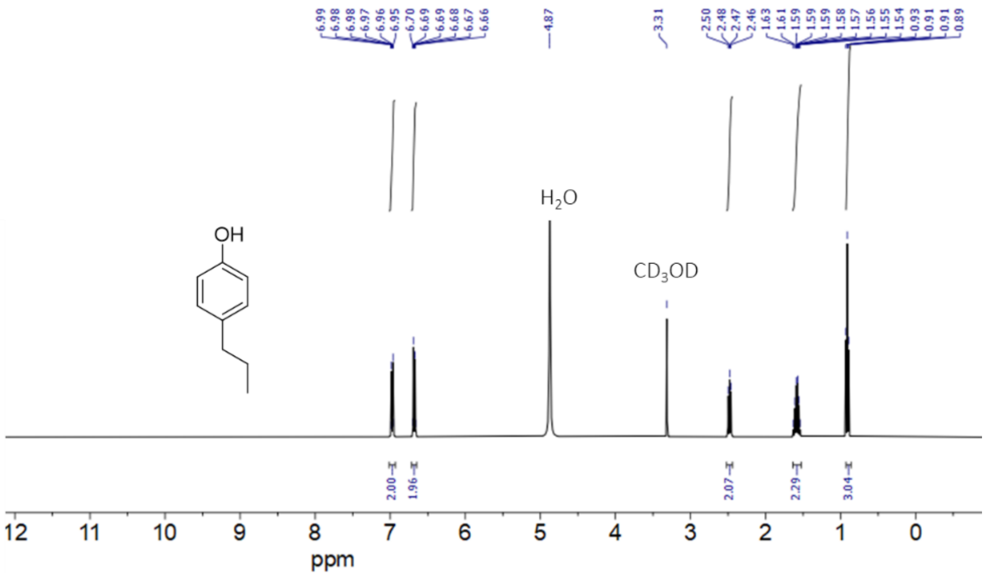

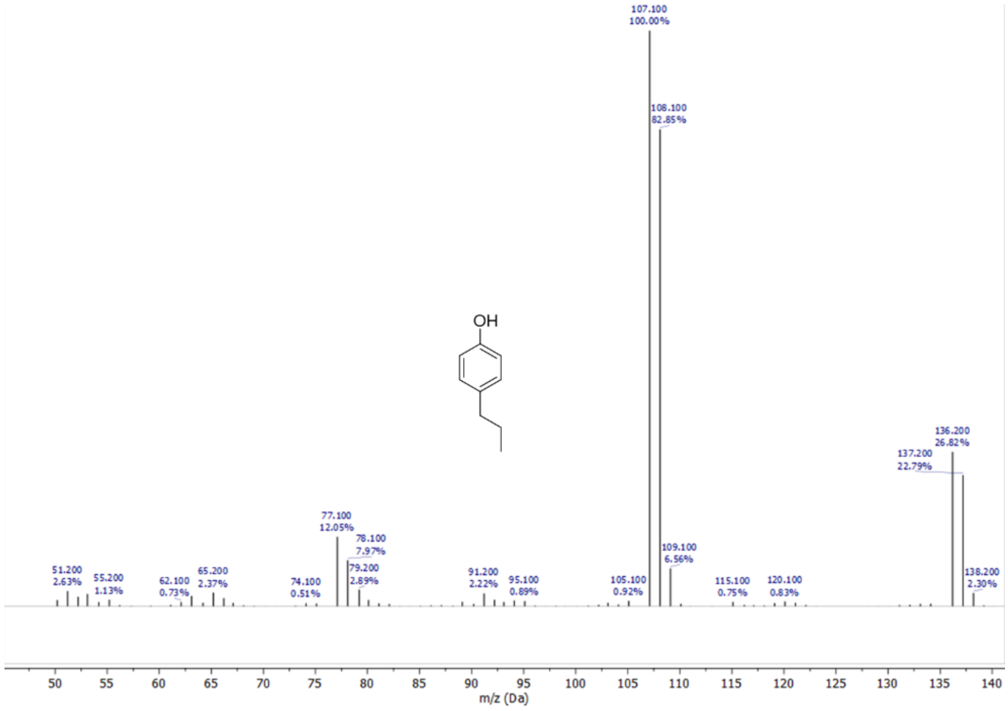

Figure S12: ^{1}H NMR (400 MHz) and mass spectra of the product from Table 2, entry 1.

Table 2, entry 2: The product was isolated as a white solid by extraction with ethyl acetate and purification by column chromatography. The obtained analytical data matched the values reported for 2,6-dimethylphenol.[25,26] ^{1}H NMR (400 MHz, MeOD) δ 6.95 (dd, *J* = 16.0, 7.3 Hz, 2H), 6.66 (t, *J* = 7.5 Hz, 1H), 2.18 (s, 6H). *m/z* calculated for [M$^+$]: 121.1; found: 122.1 (86.6%).

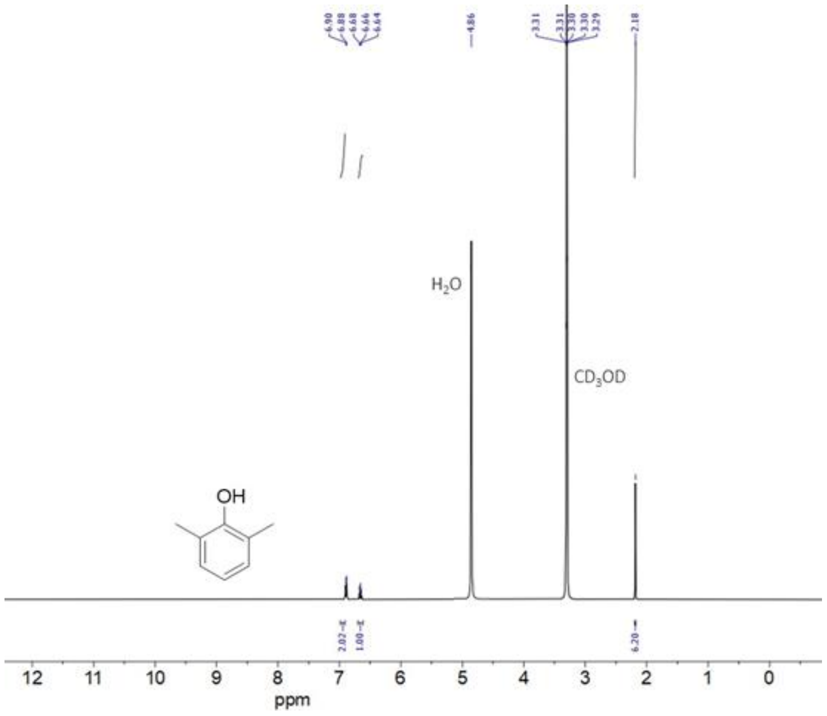

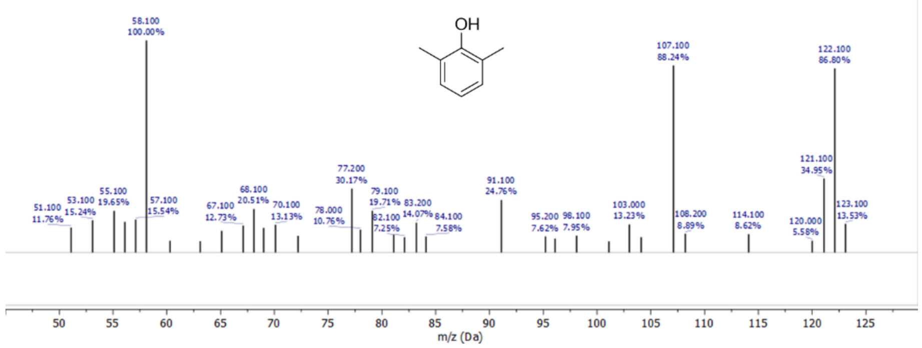

Figure S13: ^{1}H NMR (400 MHz, MeOD) and mass spectra of the product from Table 2, entry 2.

Table 2, entry 3: The product was isolated as a white solid by extraction with ethyl acetate and purification by column chromatography. The obtained analytical data matched the values reported for 4-hydroxybenzaldehyde.[24] ^{1}H NMR (400 MHz, MeOD) δ 9.86 (s, 1H), 7.7 (d, J = 8.4 Hz, 2H), 6.9 (d, J = 9.0 Hz, 2H), 6.73 (s, 1H). ^{13}C NMR (101 MHz, MeOD) δ 192.6, 165.3, 133.2, 130.0, 116.7. *m/z* calculated for [M^{+}]: 122.1; found: 122.1.

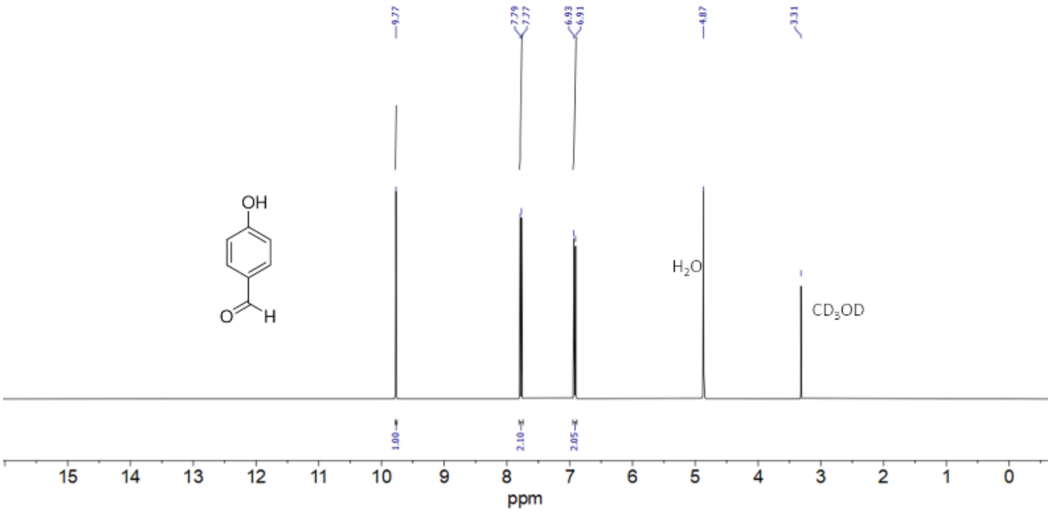

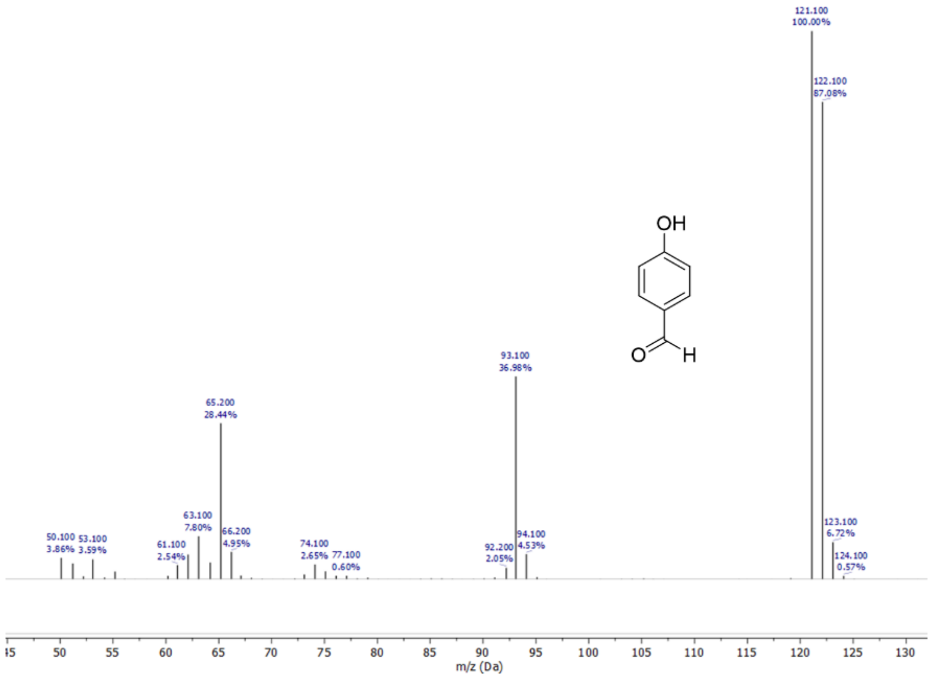

Figure S14: ^{1}H NMR (400 MHz, MeOD) and mass spectra of the product from Table 2, entry 3.

Table 2, entry 4: The product was isolated as a white solid by extraction with ethyl acetate and purification by column chromatography. The obtained analytical data matched the values reported for 4-(hydroxymethyl)phenol.[26,27] ^{1}H NMR (400 MHz, MeOD) δ 7.17 (d, *J* = 8.5 Hz, 2H), 6.75 (d, *J* = 8.5 Hz, 2H), 4.48 (s, 2H). *m/z* calculated for [M$^+$]: 124.1; found: 124.1.

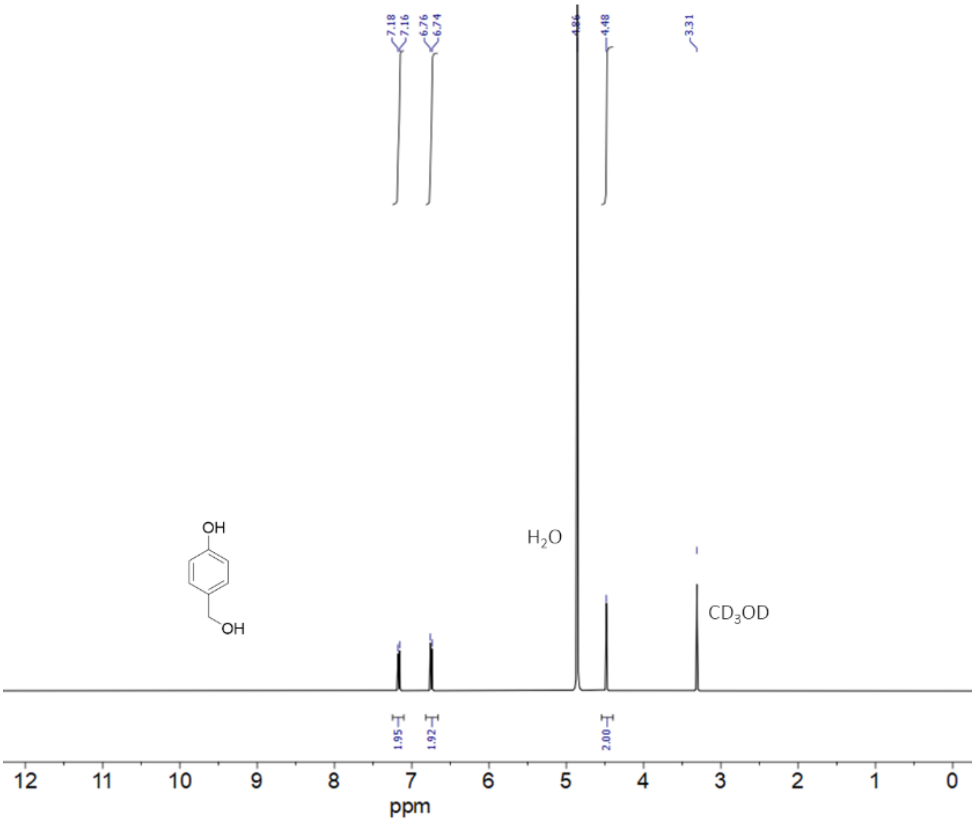

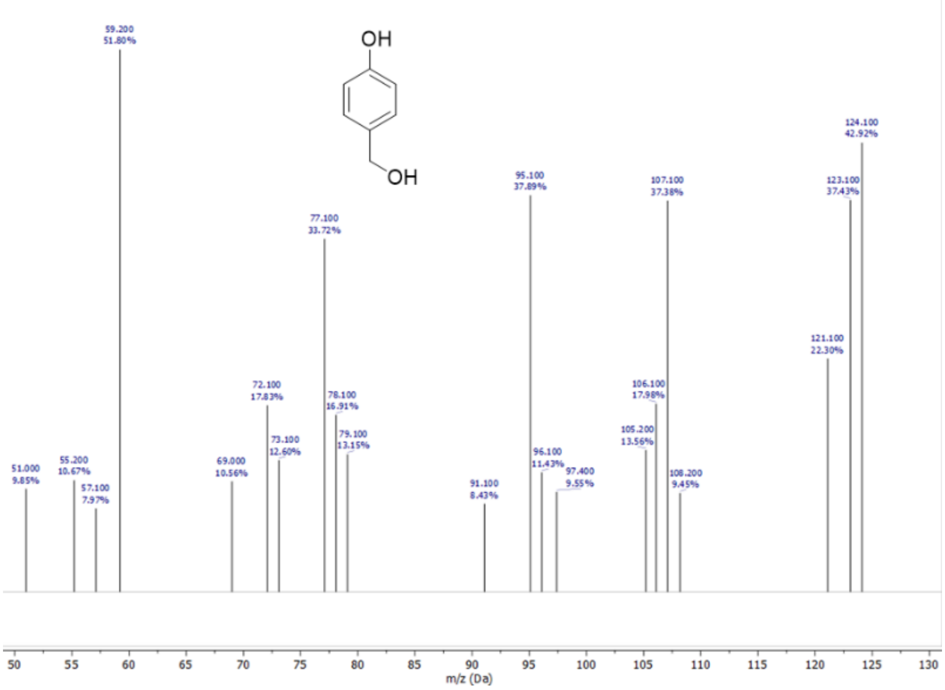

Figure S15: ^{1}H NMR (400 MHz, MeOD) and mass spectra of the product from Table 2, entry 4.

Table 2, entry 5: The product was isolated as a white solid by extracting the slightly acidic solution with ethyl acetate and chloroform. The obtained analytical data matched the values reported for 4-hydroxybenzoic acid.[24] ^{1}H NMR (400 MHz, MeOD) δ 7.83 (d, J = 8.8 Hz, 2H), 6.74 (d, J = 8.7 Hz, 2H). m/z calculated for $[M^{+}]$: 138.1; found: 138.1.

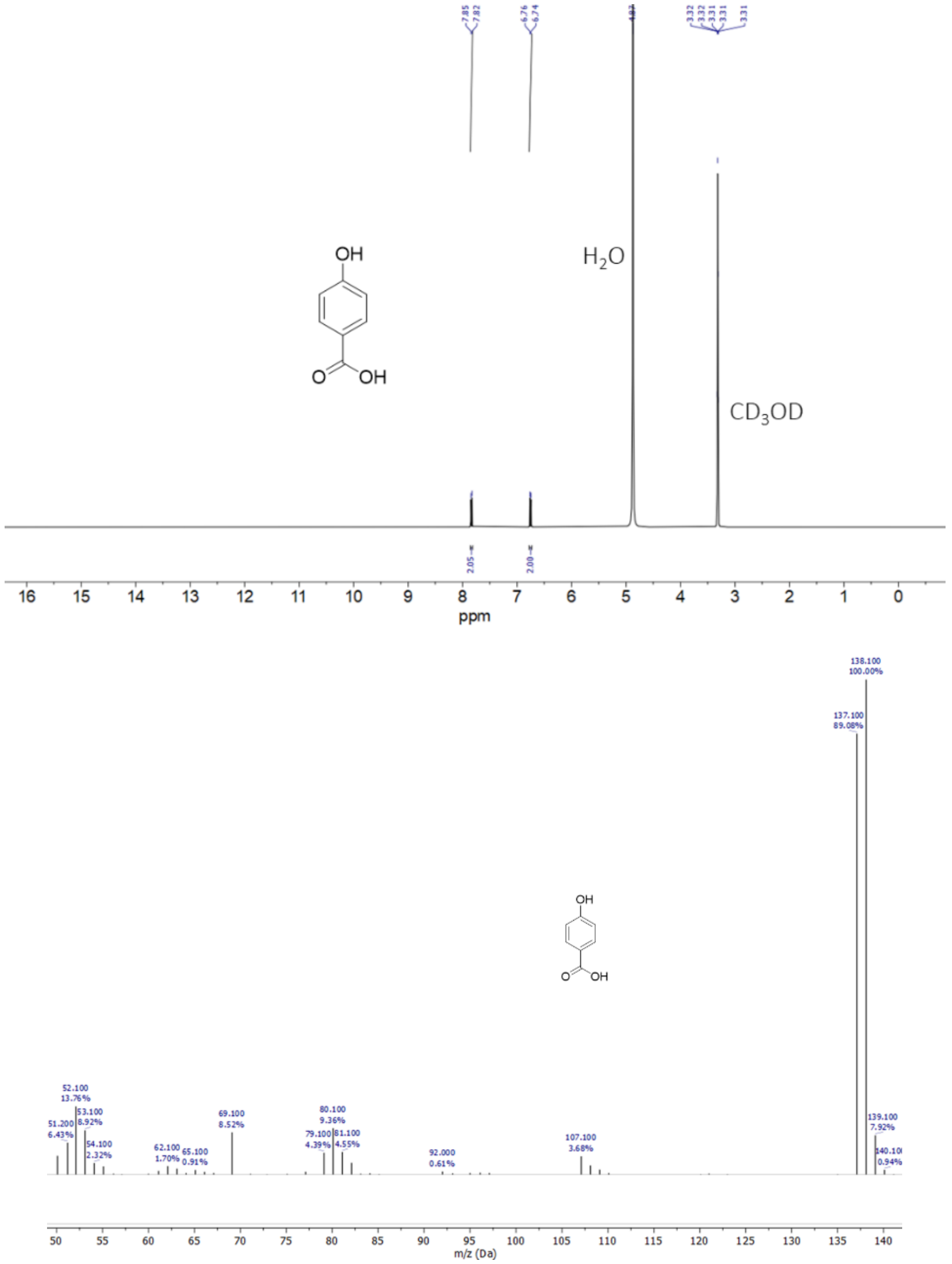

Figure S16: ^{1}H NMR (400 MHz, MeOD) and mass spectra of the product from Table 2, entry 5.

Table 2, entry 6: The product was isolated as a yellow solid by extraction with ethyl acetate and purification by column chromatography. The obtained analytical data matched the values reported for 4-nitrophenol.[24] ^{1}H NMR (400 MHz, MeOD) δ 8.11 (s, 2H), 6.89 (d, *J* = 9.1 Hz, 2H). *m/z* calculated for [M$^+$]: 139.1; found: 139.1.

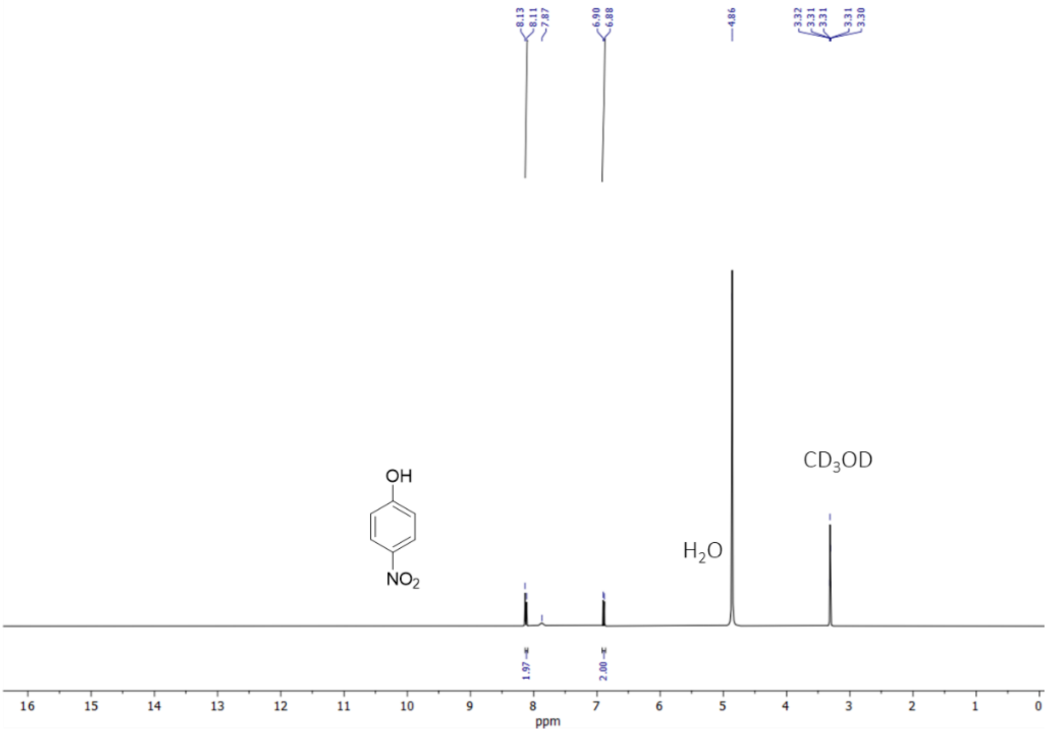

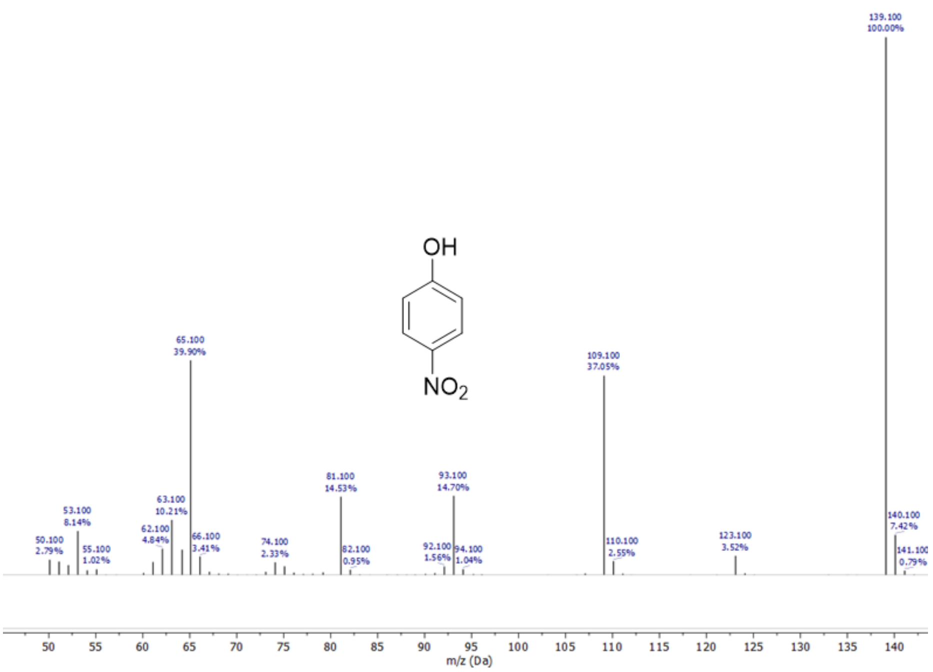

Figure S17: ^{1}H NMR (400 MHz, MeOD) and mass spectra of the product from Table 2, entry 6.

Table 2, entry 7: The product was isolated as a yellow solid by extraction with ethyl acetate and purification by column chromatography. The obtained analytical data matched the values reported for 4-fluorophenol.[27] ^{1}H NMR (400 MHz, $CDCl_3$) δ 6.95 – 6.88 (m, 2H), 6.80 – 6.74 (m, 2H). *m/z* calculated for [M$^+$]: 112.1; found: 112.1 (100%).

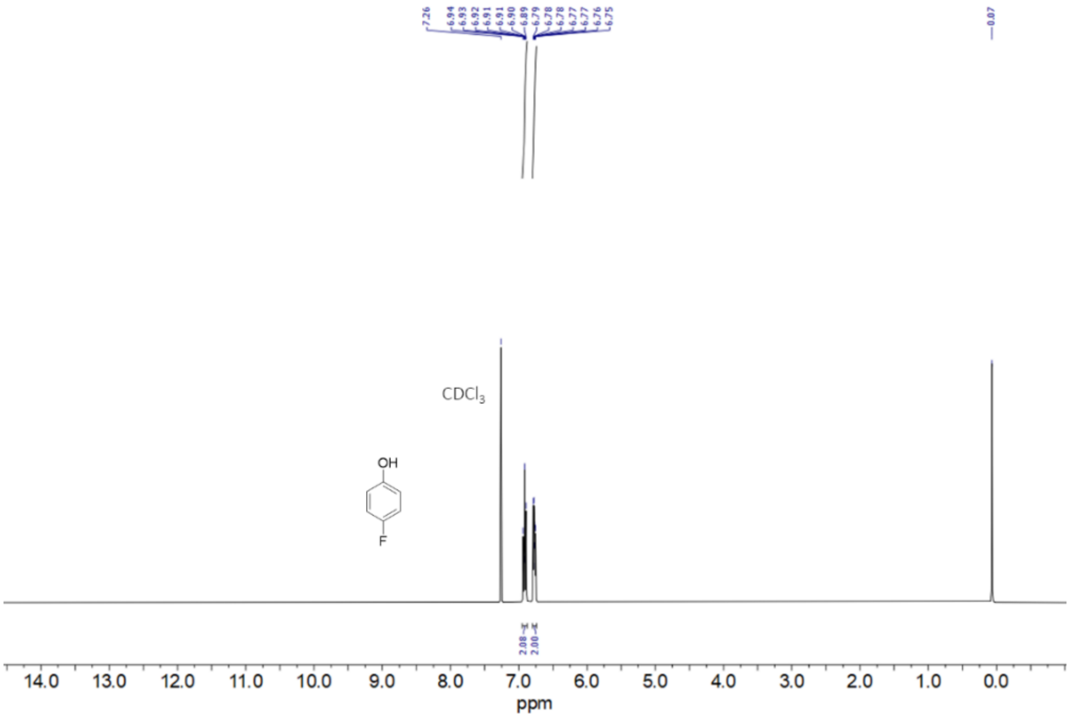

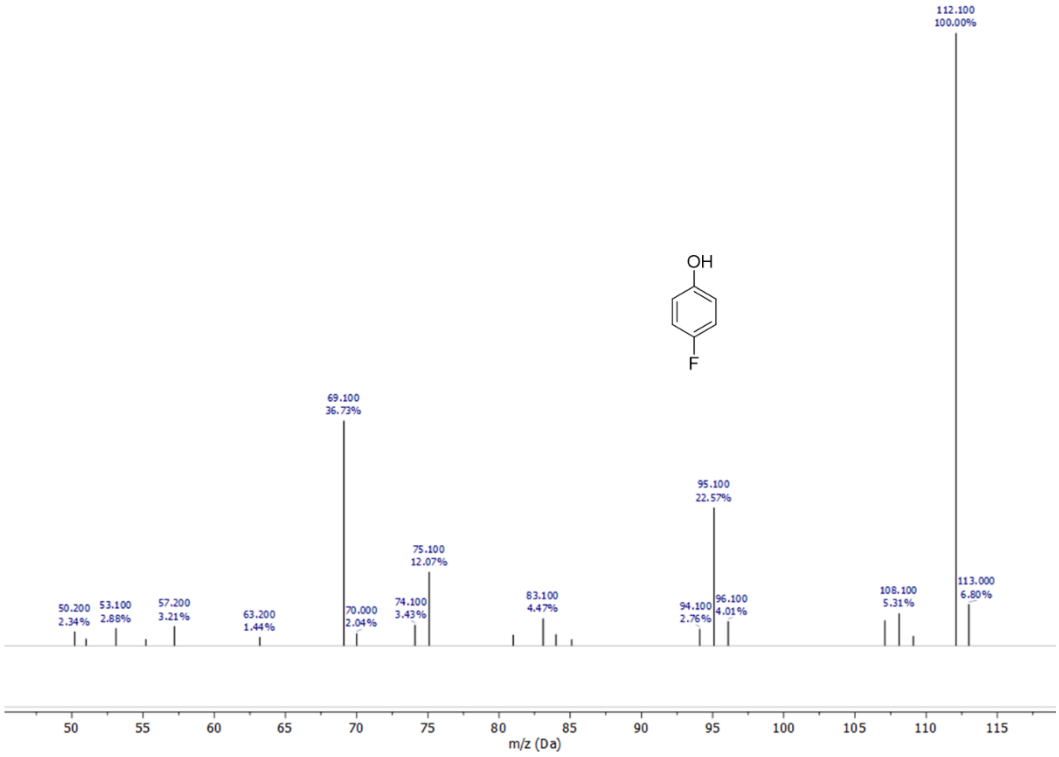

Figure S18: ^{1}H NMR (400 MHz, MeOD) and mass spectra of the product from Table 2, entry 7.

Table 2, entry 8: The product was isolated as a light orange solid by extraction with ethyl acetate and purification by column chromatography. The obtained analytical data matched the values reported for 2-fluoro-4-methoxyphenol.[28] ^{1}H NMR (400 MHz, MeOD) δ 7.05 (d, *J* = 4.3 Hz, 1H), 6.85 (dd, *J* = 11.1, 8.9 Hz, 1H), 6.42 (dd, *J* = 7.6, 3.0 Hz, 1H), 6.17 (dt, *J* = 8.9, 3.2 Hz, 1H), 3.70 (s, 3H). *m/z* calculated for [M^{+}]: 142.1; found: 142.1.

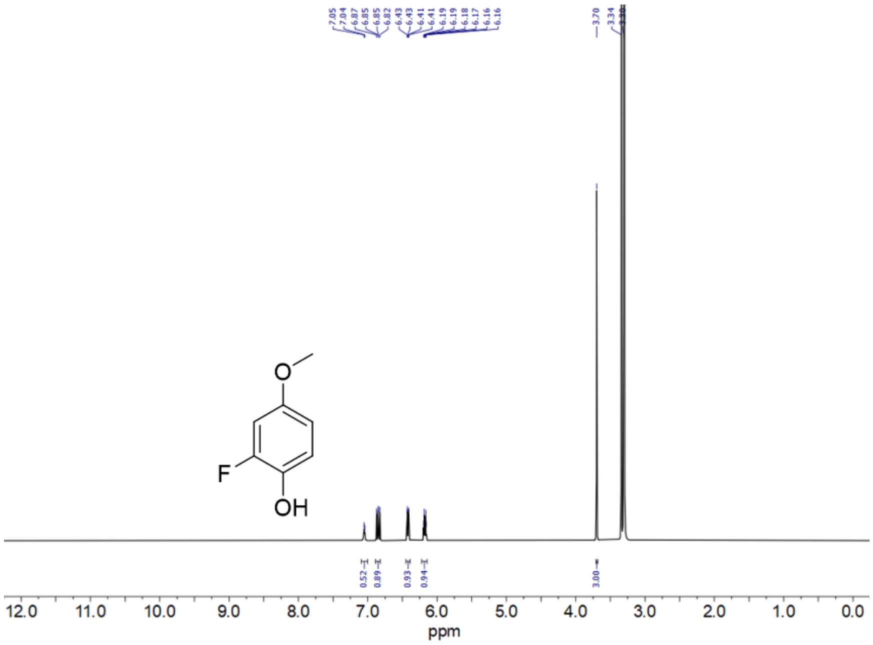

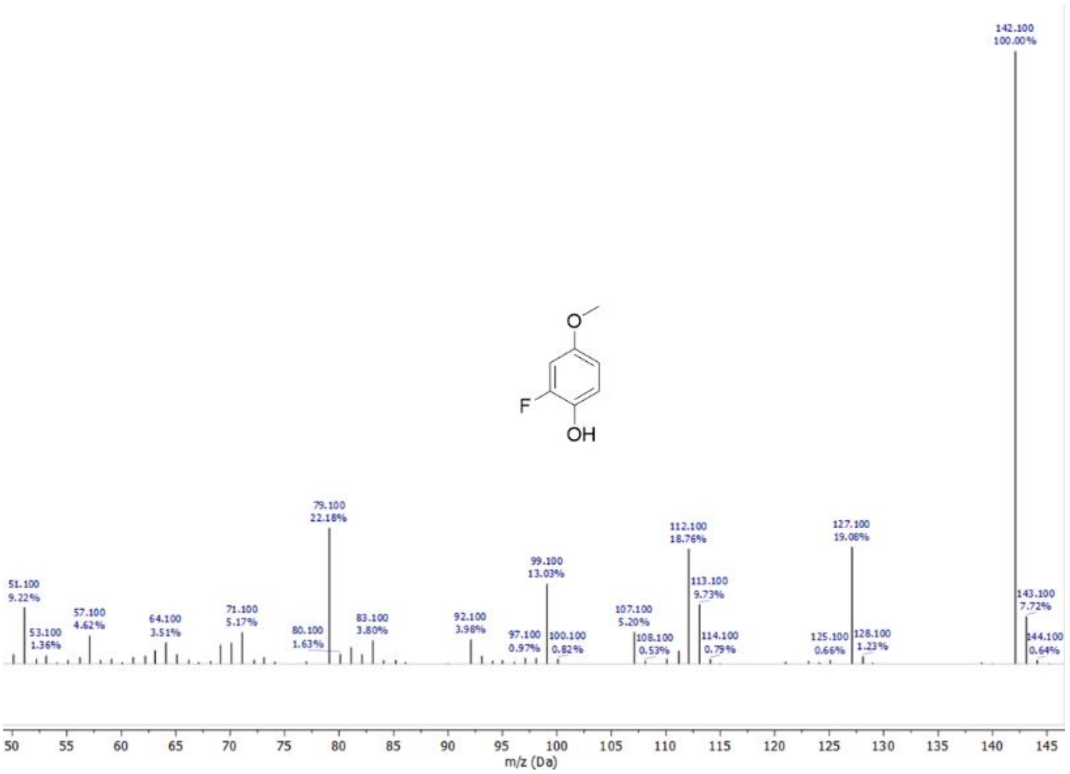

Figure S19: ^{1}H NMR (400 MHz, MeOD) and mass spectra of the product from Table 2, entry 8.

Table 2, entry 9: The product was isolated as a light-yellow solid by extraction with ethyl acetate and purification by column chromatography. The obtained analytical data matched the values reported for 3-hydroxy-4-methoxy-benzaldehyde.[29,30] ^{1}H NMR (400 MHz, MeOD) δ 9.76 (s, 1H), 7.43 (dd, *J* = 8.3, 2.0 Hz, 1H), 7.30 (d, *J* = 2.0 Hz, 1H), 7.10 (d, *J* = 8.3 Hz, 1H), 3.95 (s, 3H). *m/z* calculated for [M$^+$]: 152.1 and 153.1; found: 151.1 (100%), 152.1 (92.4%), and 153.1 (8.4%).

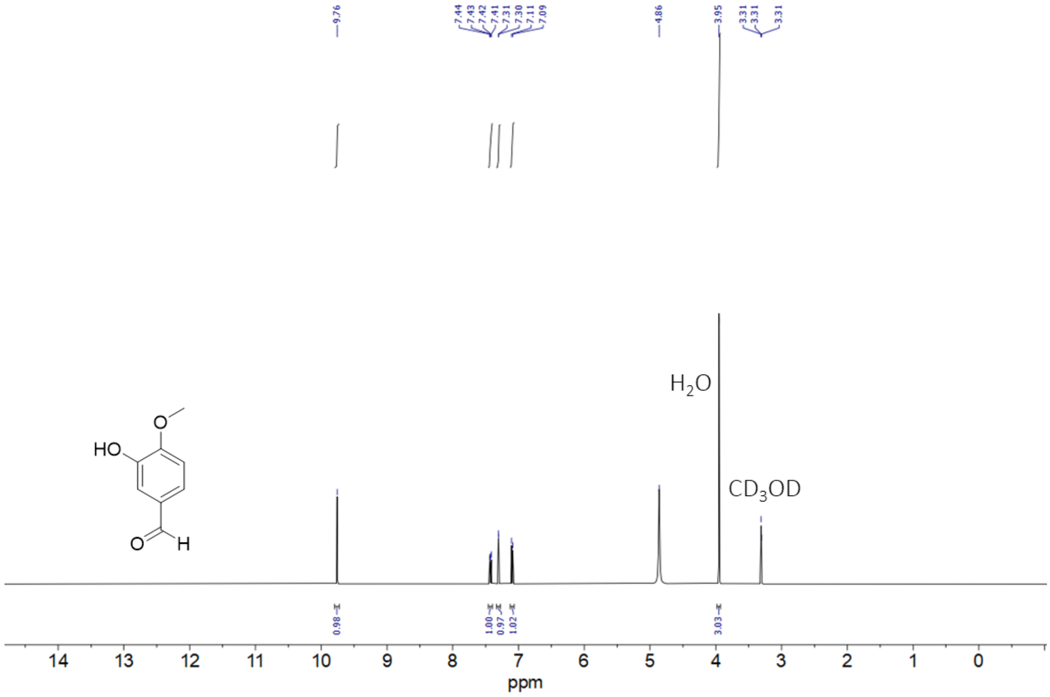

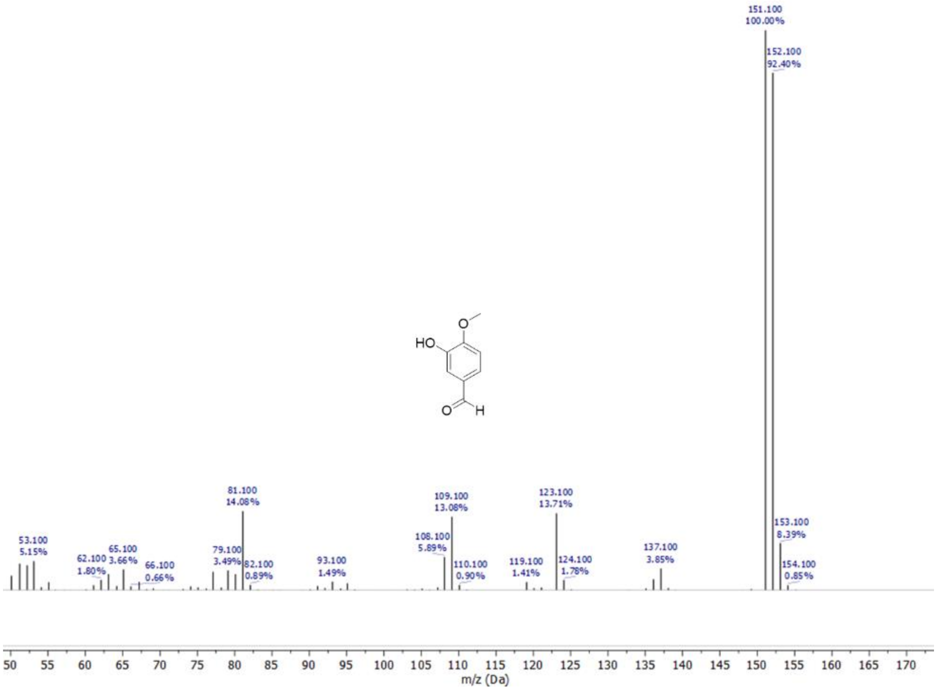

Figure S20: ^{1}H NMR (400 MHz, MeOD) and mass spectra of the product from Table 2, entry 9.

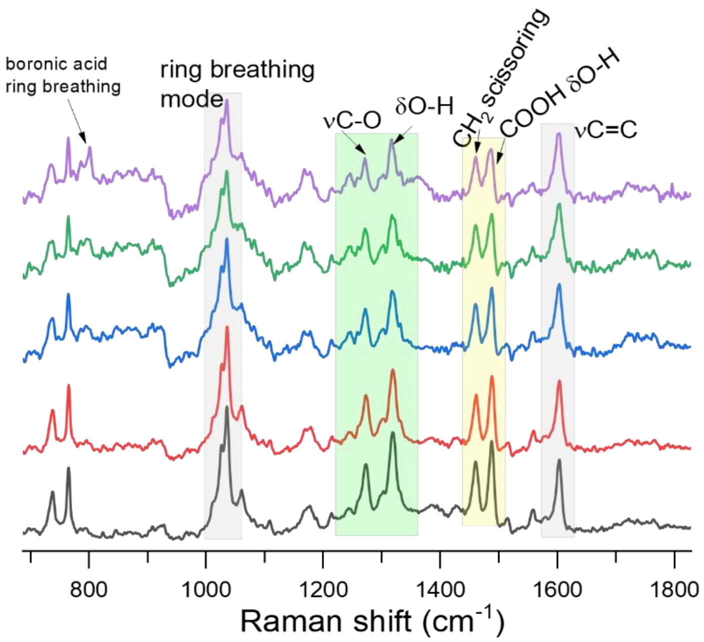

Figure S21: Raman spectrum of PDha-*co*-(APTMA-co-Ru) (black) and PDha-*co*-(APTMA-*co*-Ru)/4-MPBA with different ratio (red: 5wt%, blue 10 wt.%, green 20 wt.% and purple 40 wt.%) obtained during passive dehydration of the samples.

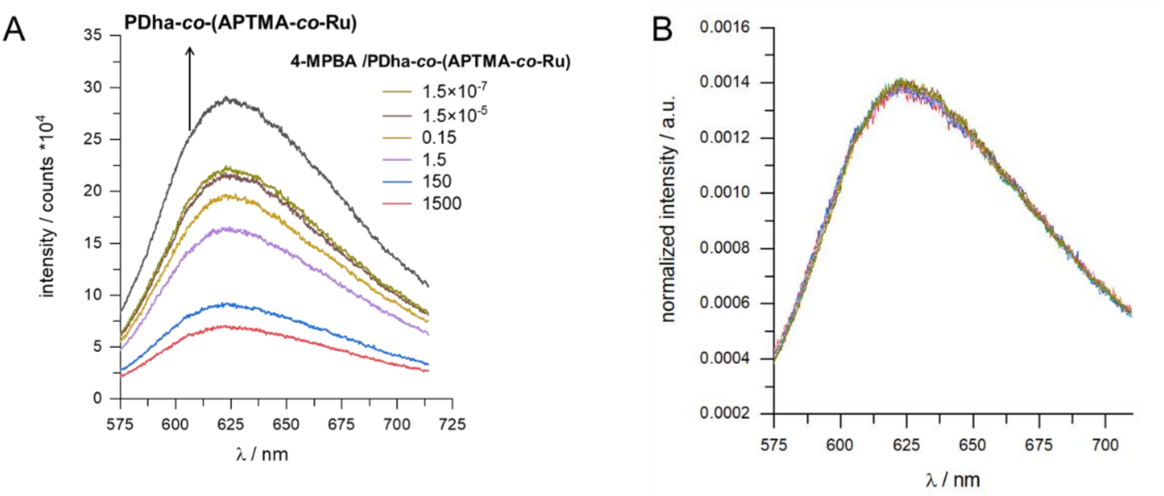

Figure S22: (A) Time-resolved emission spectra of 3 mL solutions of PDha-*co*-(APTMA-*co*-Ru) (~6.6 µg/mL) and solution containing different ratios of 4-MPBA to PDha-*co*-(APTMA-*co*-Ru), collected at a gate delay of Δt = 75 ns after the laser pulse with excitation at λ_{ex} = 450 nm. (B) Area-normalized emission spectra (data from A).

Table S7: Emission kinetic data for solutions of PDha-*co*-(APTMA-*co*-Ru) (~6.6 µg/mL) containing different wt/wt ratios of 4-MPBA to PDha-*co*-(APTMA-*co*-Ru). In the data analysis the luminescence decay times τ_1 and τ_2 were set a global parameters and only the respective relative amplitudes (A1, A2 (%)) were fitted for each luminescence decay kinetics individually.

Sample		τ1 (ns)	A1 (%)	τ2 (ns)	A2(%)
PDha-*co*-(APTMA-*co*-Ru)		244	33	814	67
4-MPBA/ PDha-*co*-(APTMA-*co*-Ru)					
	(wt/wt)				
	1.5×10^{-7}		17		83
	1.5×10^{-5}		25		75
	1.5×10^{-1}		23		77
	1.5×10^{0}		37		63
	1.5×10^{1}		41		59
	1.5×10^{2}		60		40

The amplitude-averaged luminescence decay time was obtained from individually fitting each luminescence decay kinetics with a bi-exponential decay law:

$$I(t) = A1 \exp(-t/\tau 1) + A2 \exp(-t/\tau 2) + \text{const.}$$

Cyclic voltammetry results

The model complex [Ru(bpy)$_2$(phen-dione)]Cl$_2$ displays a pH-independent anodic wave at positive potential corresponding to the Ru(II/III) couple and two cathodic peaks at negative potentials attributed to sequential one-electron reductions of the coordinated phen-dione to semiquinone and diolate.[31] These redox processes exhibit scan rate-dependent behavior, consistent with diffusion-controlled electron transfer. No bipyridine-based, and Ru(II/I) reductions are observed due to their occurrence beyond the water electrolysis window.

In contrast, the PDha-*co*-(APTMA-*co*-DHPH) copolymer bearing a redox-active 2-hydroxyphenyl-hydrazone (DHPH) moiety exhibits a single, irreversible cathodic peak in aqueous solution (Figure 4B), attributed to the one-electron reduction of the phenol, and NH group in the structure in the absence of metal coordination. Upon incorporation of Ru(II) into the polymer matrix, voltammetric features change significantly: the cathodic peak associated with the phenanthroline unit disappears, and anodic peak assigned to the Ru(II/III) oxidation appears at a more positive potential compared to the model complex, that confirms successful complexation of Ru to the polymer-bound ligand. The suppression of the cathodic process upon coordination likely arises from altered electron distribution, increased conjugation, and restricted accessibility of the redox-active site within the macromolecular environment. These results collectively support the formation of a Ru–polymer coordination complex and illustrate how metal–ligand interactions and matrix effects modulate the redox behavior of functionalized polymeric systems.[32–34]

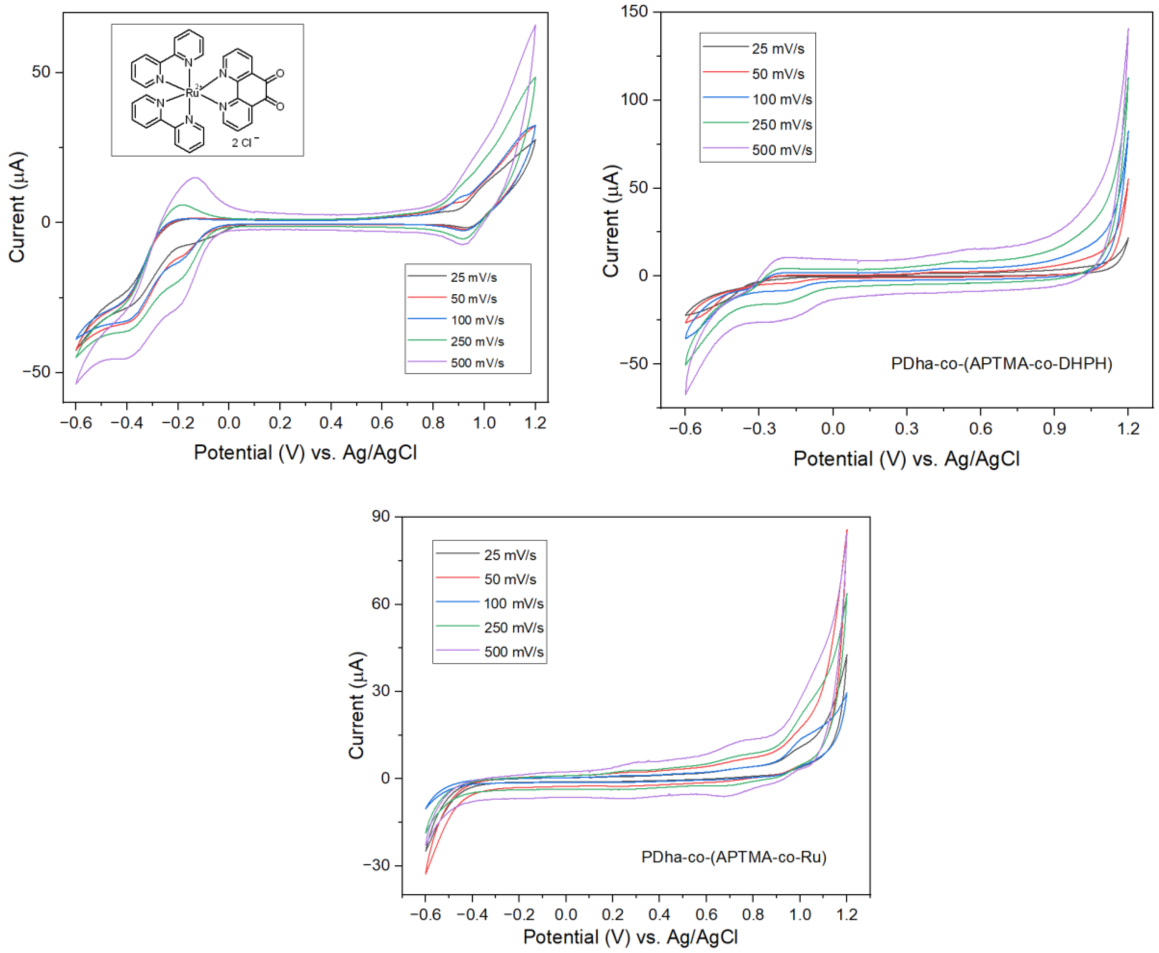

Figure S23: Cyclic voltammograms of aqueous solutions of [Ru(bpy)$_2$(phen-dione)]Cl$_2$, PDha-*co*-(APTMA-*co*-DHPH) and PDha-co-(APTMA-co-Ru) copolymers -KCl (100 mM) on SPCE (various scan rates, potential range: −0.6 to +1.2 V vs. Ag/AgCl.

References:

(1) Harrisson, S.; Couvreur, P.; Nicolas, J. Simple and Efficient Copper Metal-Mediated Synthesis of Alkoxyamine Initiators. *Polymer Chemistry* **2011**, *2* (8), 1859–1865.

(2) Grimaldi, S.; Finet, J. P.; Le Moigne, F.; Zeghdaoui, A.; Tordo, P.; Benoit, D.; Fontanille, M.; Gnanou, Y. Acyclic β-Phosphonylated Nitroxides: A New Series of Counter-Radicals for 'Living'/Controlled Free Radical Polymerization. *Macromolecules* **2000**, *33* (4), 1141–1147.

(3) Gigmes, D.; Vinas, J.; Chagneux, N.; Lefay, C.; Phan, T. N. T.; Trimaille, T.; Dufils, P.-E.; Guillaneuf, Y.; Carrot, G.; Boué, F.; Bertin, D. SG1 and BLOCBUILDER® Technology: A Versatile Toolbox for the Elaboration of Complex Macromolecular Architectures. In *ACS Symposium Series*; ACS Publications, 2009; Vol. 1024, pp 245–262.

(4) Dumur, F.; Guerlin, A.; Lehoux, A.; Selvakannan, P. R.; Miomandre, F.; Méallet-Renault, R.; Rebarz, M.; Sliwa, M.; Dumas, E.; Le Pleux, L.; Pellegrin, Y.; Odobel, F.; Mayer, C. R. Mutual Influence of Gold and Silver Nanoparticles on Tris-(2,2′bipyridine)-Ru(II) Core Complexes: Post-Functionalization Processes, Optical and Electrochemical Investigations. *Applied Surface Science* **2020**, *499*, 143847.

(5) Hidayatullah, A. N.; Wachter, E.; Heidary, D. K.; Parkin, S.; Glazer, E. C. Photoactive Ru(II) Complexes With Dioxinophenanthroline Ligands Are Potent Cytotoxic Agents. *Inorganic Chemistry* **2014**, *53* (19), 10030–10032.

(6) Dumur, F.; Mayer, C. R.; Hoang-Thi, K.; Ledoux-Rak, I.; Miomandre, F.; Clavier, G.; Dumas, E.; Méallet-Renault, R.; Frigoli, M.; Zyss, J.; Sécheresse, F. Electrochemical, Linear Optical, and Nonlinear Optical Properties and Interpretation by Density Functional Theory Calculations of (4-N,N- Dimethylaminostyryl)-Pyridinium Pendant Group Associated with Polypyridinic Ligands and Respective Multifunctional Me. *Inorganic Chemistry* **2009**, *48* (17), 8120–8133.

(7) Neese, F.; Wennmohs, F.; Becker, U.; Riplinger, C. The ORCA Quantum Chemistry Program Package. *Journal of Chemical Physics* **2020**, *152* (22).

(8) Neese, F. Software Update: The ORCA Program System—Version 5.0. *Wiley Interdisciplinary Reviews: Computational Molecular Science* **2022**, *12* (5), e1606.

(9) Becke, A. D. Thermochemistry. III. The Role of Exact Exchange. *J Chem Phys* **1993**, *98* (December 1992), 5648–5652.

(10) Grimme, S.; Ehrlich, S.; Goerigk, L. Effect of the Damping Function in Dispersion Corrected Density Functional Theory. *Journal of Computational Chemistry* **2011**, *32* (7), 1456–1465.

(11) Weigend, F.; Ahlrichs, R. Balanced Basis Sets of Split Valence, Triple Zeta Valence and Quadruple Zeta Valence Quality for H to Rn: Design and Assessment of Accuracy. *Physical Chemistry Chemical Physics* **2005**, *7* (18), 3297–3305.

(12) Barone, V.; Cossi, M. Quantum Calculation of Molecular Energies and Energy Gradients in Solution by a Conductor Solvent Model. *Journal of Physical Chemistry A* **1998**, *102* (11), 1995–2001.

(13) Garcia-Ratés, M.; Neese, F. Effect of the Solute Cavity on the Solvation Energy and Its Derivatives within the Framework of the Gaussian Charge Scheme. *Journal of Computational Chemistry* **2020**, *41* (9), 922–939.

(14) Neese, F. An Improvement of the Resolution of the Identity Approximation for the Formation of the Coulomb Matrix. *Journal of Computational Chemistry* **2003**, *24* (14), 1740–1747.

(15) Neese, F.; Wennmohs, F.; Hansen, A.; Becker, U. Efficient, Approximate and Parallel Hartree-Fock and Hybrid DFT Calculations. A "chain-of-Spheres" Algorithm for the Hartree-Fock Exchange. *Chemical Physics* **2009**, *356* (1–3), 98–109.

(16) Helmich-Paris, B.; de Souza, B.; Neese, F.; Izsák, R. An Improved Chain of Spheres for Exchange Algorithm. *Journal of Chemical Physics* **2021**, *155* (10).

(17) Weigend, F. Accurate Coulomb-Fitting Basis Sets for H to Rn. *Physical Chemistry Chemical Physics* **2006**, *8* (9), 1057–1065.

(18) CASIDA, M. E. Time-Dependent Density Functional Response Theory for Molecules. In *Recent Advances In Density Functional Methods: (Part I)*; World Scientific, 1995; pp 155–192.

(19) Giannoudis, E.; Bold, S.; Müller, C.; Schwab, A.; Bruhnke, J.; Queyriaux, N.; Gablin, C.; Leonard, D.; Saint-Pierre, C.; Gasparutto, D.; Aldakov, D.; Kupfer, S.; Artero, V.; Dietzek, B.; Chavarot-Kerlidou, M. Hydrogen Production at a NiO Photocathode Based on a Ruthenium Dye-Cobalt Diimine Dioxime Catalyst Assembly:

Insights from Advanced Spectroscopy and Post-Operando Characterization. *ACS Applied Materials and Interfaces* **2021**, *13* (42), 49802–49815.

(20) Titov, E.; Saalfrank, P. Exciton Splitting of Adsorbed and Free 4-Nitroazobenzene Dimers: A Quantum Chemical Study. *Journal of Physical Chemistry A* **2016**, *120* (19), 3055–3070.

(21) Costabel, D.; Skabeev, A.; Nabiyan, A.; Luo, Y.; Max, J. B.; Rajagopal, A.; Kowalczyk, D.; Dietzek, B.; Wächtler, M.; Görls, H. 1, 7, 9, 10-Tetrasubstituted PMIs Accessible through Decarboxylative Bromination: Synthesis, Characterization, Photophysical Studies, and Hydrogen Evolution Catalysis. *Chemistry–A European Journal* **2021**, *27* (12), 4081–4088.

(22) Kowalczyk, D.; Li, P.; Abbas, A.; Eichhorn, J.; Buday, P.; Heiland, M.; Pannwitz, A.; Schacher, F. H.; Weigand, W.; Streb, C. Making Photocatalysis Comparable Using a Modular and Characterized Open-Source Photoreactor. *ChemPhotoChem* **2022**, *6* (7), e202200044.

(23) Pitre, S. P.; McTiernan, C. D.; Ismaili, H.; Scaiano, J. C. Mechanistic Insights and Kinetic Analysis for the Oxidative Hydroxylation of Arylboronic Acids by Visible Light Photoredox Catalysis: A Metal-Free Alternative. *Journal of the American Chemical Society* **2013**, *135* (36), 13286–13289.

(24) Zou, Y. Q.; Chen, J. R.; Liu, X. P.; Lu, L. Q.; Davis, R. L.; Jørgensen, K. A.; Xiao, W. J. Highly Efficient Aerobic Oxidative Hydroxylation of Arylboronic Acids: Photoredox Catalysis Using Visible Light. *Angewandte Chemie - International Edition* **2012**, *51* (3), 784–788.

(25) Nakamura, R.; Obora, Y.; Ishii, Y. Selective One-Pot Synthesis of Various Phenols from Diarylethanes. *Chemical Communications* **2008**, No. 29, 3417–3419.

(26) Kotoučová, H.; Strnadová, I.; Kovandová, M.; Chudoba, J.; Dvořáková, H.; Cibulka, R. Biomimetic Aerobic Oxidative Hydroxylation of Arylboronic Acids to Phenols Catalysed by a Flavin Derivative. *Organic and Biomolecular Chemistry* **2014**, *12* (13), 2137–2142.

(27) Yu, K.; Zhang, H.; Sheng, Y.; Zhu, Y. Visible-Light-Promoted Aerobic Oxidative Hydroxylation of Arylboronic Acids in Water by Hydrophilic Organic Semiconductor. *Tetrahedron Letters* **2020**, *61* (28), 152010.

(28) Udwary, D. W.; Casillas, L. K.; Townsend, C. A. Synthesis of 11-Hydroxyl O-Methylsterigmatocystin and the Role of a Cytochrome P-450 in the Final Step of Aflatoxin Biosynthesis. *Journal of the American Chemical Society* **2002**, *124* (19), 5294–5303.

(29) Pal, M.; Parasuraman, K.; Yeleswarapu, K. R. Palladium-Catalyzed Cleavage of O/N-Propargyl Protecting Groups in Aqueous Media under a Copper-Free Condition. *Organic Letters* **2003**, *5* (3), 349–352.

(30) Annen, S.; Zweifel, T.; Ricatto, F.; Grützmacher, H. Catalytic Aerobic Dehydrogenative Coupling of Primary Alcohols and Water to Acids Promoted by a Rhodium(I) Amido N-Heterocyclic Carbene Complex. *ChemCatChem* **2010**, *2* (10), 1286–1295.

(31) Murphy, D. M.; McNamara, K.; Richardson, P.; Sanchez-Romaguera, V.; Winpenny, R. E. P.; Yellowlees, L. J. Electrochemical and Spectroelectrochemical Studies of Complexes of 1,10-Phenanthroline-5,6-Dione. *Inorganica Chimica Acta* **2011**, *374* (1), 435–441.

(32) Goss, C. A.; Abruña, H. D. Spectral, Electrochemical, and Electrocatalytic Properties of l,10-Phenanthroline-5,6-Dione Complexes of Transition Metals. *Inorganic Chemistry* **1985**, *24* (25), 4263–4267.

(33) Yokoyama, K.; Wakabayashi, A.; Noguchi, K.; Nakamura, N.; Ohno, H. Structure and Spectroelectrochemical Property of a Ruthenium Complex Containing Phenanthroline-Quinone, and Assembly of the Complexes on a Gold Electrode. *Inorganica Chimica Acta* **2006**, *359* (3), 807–814.

(34) Hickson, J. R.; Horsewill, S. J.; McGuire, J.; Wilson, C.; Sproules, S.; Farnaby, J. H. The Semiquinone Radical Anion of 1,10-Phenanthroline-5,6-Dione: Synthesis and Rare Earth Coordination Chemistry. *Chemical Communications* **2018**, *54* (80), 11284–11287.
